# Supplementary material for: Identification and Profiling of a Novel Bombyx mori latent virus Variant Acutely Infecting Helicoverpa armigera and Trichoplusia ni
Source: Viruses. 2023 May 17;15(5):1183. doi: 10.3390/v15051183 (PMC10221933; doi:10.3390/v15051183)
Supplement: Supplementary file 1 [file viruses-15-01183-s001.zip › viruses-2372211-supplementary.pdf]

Supplementary data:

Supplementary Table S1 – Virus species and sequences identifiers used for phylogenetic tree construction

Virus	Abbreviation	Genus:	Reference
Bombyx mori latent virus	BmLV	*Maculavirus*	AB624361.1
Bombyx mori latent virus	BmLV	*Maculavirus*	AB186123.1
Bombyx mori latent virus	BmLV	*Maculavirus*	KJ433990.1
grapevine fleck virus	GFkV	*Maculavirus*	NP_542612.1
grapevine red globe virus	GRGV	*Maculavirus*	YP_009268923.1
bee macula-like virus 2	BeeMLV2	*Maculavirus*	YP_009551952.1
turnip yellow mosaic virus	TYMV	*Tymovirus*	NP_663297.1
eggplant mosaic virus	EMV	*Tymovirus*	NP_040968.1
anagyris vein yellowing virus	AVYV	*Tymovirus*	YP_002308578.1
oat blue dwarf virus	OBDV	*Marafivirus*	NP_044447.1
maize rayado fino virus	MRFV	*Marafivirus*	NP_115454.2
citrus sudden death-associated virus	CSDaV	*Marafivirus*	YP_224218.1
potato virus x	PVX	*Potexvirus*	YP_002332929.1

Supplementary Table S2 – Composition of *Helicoverpa* artificial diet

**Component:**	**Weight (g):**	**Brand:**
Wheat Germ	72	Merck
Casein	33	Merck
Sugar	29.3	Generic
Brewer's Yeast	14.3	Generic
Wesson's salt	9.4	MP Biomedicals
Sorbic acid	1.5	Merck
4-hydroxybenzoic acid	0.93	Merck
linseed oil	1.87	MP Biomedicals
Bactoagar	18.8	BD
Vanderzant vitamin mix	1.0	Merck
Streptomycin sulphate	1.1	Merck
L-ascorbic acid	4.4	Merck
Methyl Paraben	2	Merck
cholesterol	0.5	Merck
water	To 1 liter	

Supplementary Table S3 – List of PCR primers

Purpose	Species	Gene (GenBank or RefSeq)	Strand	Sequence	Reference
qPCR	*Bombyx mori latent virus*	*coat protein* (de novo)	Forward	TTGACCTTTGTTGGACTACTGCTG	1
			Reverse	GTGGTTGGTGTATGGAATCGG	
		replicase (de novo)	Forward	CGTCCTCCCAACTTGTTTGT	
			Reverse	CTCCATTTTCTTCCGTTCCA	
	Trichoplusia ni	*ribosomal protein S18* (XM_026882096.1)	Forward	GCTCTGTATCGCCGTAACCA	2
			Reverse	TGCCGTTTCGAACACAAGAC	
		α-tubulin (XM_026877842.1)	Forward	TTACCGAGTTCCAGACCAAC	
			Reverse	AAGCAGGCGTTGGTGATCTC	
		18S (KY514086)	Forward	TGAGAAACGGCTACCACATCC	3
			Reverse	GATTACGAGGCCTCGTAAGAG	
		argonaute-2 (XM_026877347.1)	Forward	GACTGCGCGTCGTATAAACC	4
			Reverse	TGACATTGCCGTCCCTCAAA	
		siwi (XM_026876665.1)	Forward	CGGCTCCAATGGTTGACCTA	2
			Reverse	TGTTAGCAGCCAGGAATGCA	
		dicer-2 (XM_026878152.1)	Forward	CGGTGACTTGCAGACTGTCT	4
			Reverse	TTCACTGGCTGTTGCGGTAT	
	Helicoverpa armigera	*elongation factor 1α* (XM_021329970.2)	Forward	GAAGTCAAGTCCGTGGAGATG	5
			Reverse	GACCTGTGCTGTGAAGTCG	
		ribosomal protein L27 (XM_021344525.2)	Forward	ACAGGTATCCCCGCAAAGTGC	5
			Reverse	GTCCTTGGCGCTGAACTTCTC	
		α-tubulin (XM_021329536.2)	Forward	CGCCCTCAACGTCGATCTAA	
			Reverse	CCAGAGGGAAGTGGATACGC	

		argonaute-2 (XM_049840284.1)	**Forward**	CGCTCTGCCGAGGAAGATAT	
			**Reverse**	CACCTGAGCGAACTGTCCTT	
		siwi (XM_049840316.1)	**Forward**	GATCGTGCTGTGCCTACTCA	
			**Reverse**	AGGTATTTCCACTCGCCACG	
		dicer-2 (XM_049847753.1)	**Forward**	GTCGTTGATCACCCAATGCG	6
			**Reverse**	TTTTCCTTCGGGCAGACCTC	
Interspecies qPCR	*Helicoverpa armigera* & *Trichoplusia ni*	*elongation factor 1α* (XM_021329970.2 and XM_026883053.1)	**Forward**	TCAACCACCCCGGTCAAATC	
			**Reverse**	TGATTTCGGCGAACTTGCAG	
		heat shock protein 90 (XM_021341131.2 and XM_026875446.1)	**Forward**	ATCCCGCATGAAGGAGAACC	
			**Reverse**	CTCATCAATGGGCTCGGTCA	
		glyceraldehyde-3-phosphate dehydrogenase (JF417983.1 and XM_026892236.1)	**Forward**	GTACGACAACGAGTTCGGCT	
			**Reverse**	TGGTCTGGATGTACTTGATGAGA	
Cloning	*Trichoplusia ni*	*argonaute-2* (XM_026877347.1)	**Forward**	TCAAGGCTGGTCGTCAGTTC	
			**Reverse**	ATTGCCCCTCTGAAACTCCG	
	Bombyx mori latent virus	Genome construct 1 (de novo)	**Forward**	CGATCAACAAAGTCAGCTTGTCC	
			**Reverse**	TTGGCGAGATTATCCCAGGC	
		Genome construct 2 (de novo)	**Forward**	GCTGGTGTACTCTCTCGAAGG	
			**Reverse**	CTCAGGGTCGCAACATTTGG	
		Genome construct 3 (de novo)	**Forward**	CTTCCCCGATTCAGGCAACT	
			**Reverse**	TTTCCCAGGTTGAGACTCGC	
		Genome construct 4 (de novo)	**Forward**	GTGCAGCTCATTCACATTACTGG	
			**Reverse**	AGAGGTGTCATTAGCAGGTAGTGG	
		Genome construct 5 (de novo)	**Forward**	TACTCTCGTCGCCACAACTC	
			**Reverse**	ACAGCAGTGTACCGCCAGTC	

		Genome construct 6 (de novo)	**Forward**	ACCTCCAGTCTTCAGCCATCC	
			**Reverse**	AGTGGTGATGTCGTACTCGCT	
		Genome construct 7 (de novo)	**Forward**	TCATGTCTGCAATGACTATACTGC	
			**Reverse**	TTGGCTAGACAGAACAACGC	
		Genome construct 8 (de novo)	**Forward**	TTACAGCGAGTACGACATCACC	
			**Reverse**	GCGAACTCCTTCTAGTACGGC	
		Genome construct 9 (de novo)	**Forward**	CATTCCGTCCTTGGTCAATGC	
			**Reverse**	AGAGCGCGATGAAACTAACAACC	
Colony PCR	*Trichoplusia ni*	*argonaute-2* (XM_026877347.1)	**Forward**	GCTCAAGGTGGTAGCCAGTT	
			**Reverse**	ACAAGCAGTTCAGATGCGGA	
	Bombyx mori latent virus	pCR4-TOPO plasmids	**Forward**	GTAAAACGACGGCCAG	
			**Reverse**	CAGGAAACAGCTATGAC	

1. Innami K, Aizawa T, Tsukui T, et al. Infection studies of nontarget mammalian cell lines with Bombyx mori macula-like virus. *J Virol Methods*. 2016;229:24-26. doi:10.1016/j.jviromet.2015.12.002
2. Santos D, Verdonckt T-W, Mingels L, et al. PIWI Proteins Play an Antiviral Role in Lepidopteran Cell Lines. *Viruses*. 2022;14(7):1442. doi:10.3390/V14071442
3. Zhao Z, Wang L, Yue D, et al. Evaluation of reference genes for normalization of RT-qPCR gene expression data for trichoplusia ni cells during antheraea pernyi (Lepidoptera: Saturniidae) Multicapsid Nucleopolyhedrovirus (AnpeNPV) infection. *J Insect Sci*. 2019;19(1). doi:10.1093/jisesa/iey133
4. Santos D, Wynant N, Van den Brande S, et al. Insights into RNAi-based antiviral immunity in Lepidoptera: acute and persistent infections in Bombyx mori and Trichoplusia ni cell lines. *Sci Rep*. 2018;8(1):2423. doi:10.1038/s41598-018-20848-6
5. Zhang S, An S, Li Z, et al. Identification and validation of reference genes for normalization of gene expression analysis using qrt-pcr in helicoverpa armigera (lepidoptera: Noctuidae). *Gene*. 2015;555(2):393-402. doi:10.1016/j.gene.2014.11.038
6. Verdonckt T-W, Vanden Broeck J. Methods for the Cost-Effective Production of Bacteria-Derived Double-Stranded RNA for in vitro Knockdown Studies. *Front Physiol*. 2022;13. doi:10.3389/fphys.2022.836106

Supplementary Table S4 – BioProjects from which SRA experiments were used for the identification of BmLV prevalence

Species	Tissue / cell line	Project	Viruses identified
H. armigera	Whole body	PRJNA261330	HaSNPV, HaIV
"	Adult gonads	PRJNA613606	N/A
B. mori	Whole body	PRJNA339876	BmNPV
"	"	PRJNA431156	N/A
"	"	PRJNA119935	BmIV
"	Eggs	PRJNA377550	N/A
"	"	PRJNA336361	N/A
"	Midgut	PRJEB7502	BmCPV
"	Hemolymph	PRJNA209084	BmIV
"	Silk Gland	PRJNA119935	BmIV
"	Ovary	PRJDB5508	N/A
"	*Bm*N4	PRJDB2257	**BmLV**, SfR
"	"	PRJDB4053	**BmLV**
"	"	PRJDB5376	**BmLV,** BmIV
"	"	PRJDB2929	**BmLV**, SfR
"	"	PRJNA383127	**BmLV**, SfR
"	"	PRJNA419912	**BmLV**, BmIV, SfRV, OpMNPV
"	"	PRJNA251635	**BmLV**, SfRV, BmIV
"	"	PRJNA251557	**BmLV**
"	"	PRJNA239665	**BmLV**
"	"	PRJNA284571	**BmLV**, SfRV
"	"	PRJNA632342	**BmLV**, TnTED
"	"	PRJDB5927	**BmLV**, SfRV, BmIV
"	"	PRJDB5142	**BmLV**, SfRV
"	"	PRJDB10429	**BmLV**, SfRV, BmIV
"	*Bm*-VF	PRJDB5144	**BmLV**, BmIV
S. frugiperda	Gonads + soma	PRJNA386859	TnTED
"	Whole body	PRJNA432886	OsEV, SeIV, SfRV
"	*Sf*-9	PRJNA523298	AcMNPV, PnPV, SfRV
H. melpomene	Whole body	PRJNA386859	HeIV
P. xylostella	Gonads	PRJNA386859	N/A

"	"	PRJNA421581	N/A
T. ni	Hi5 cells	PRJNA336361	FHV, BBV
"	"	PRJNA789788	**BmLV**, FHV
"	Gonads + soma	PRJNA336361	TnTED
O. furnacalis	"	PRJNA438858	N/A
G. mellonella	"	PRJNA510393	N/A
P. Xuthus	"	PRJNA445942	CTV

**Abbreviation**	**Virus name**
AcMNPV	Autographa californica multiple nucleopolyhedrovirus
BBV	Black Beetle Virus
BmCPV	Bombyx mori cytoplasmic polyhedrosis virus
BmIV	Bombyx mori iflavirus
BmNPV	Bombyx mori nucleopolyhedrovirus
CTV	citrus tristeza virus
FHV	Flock House Virus
GRLDaV	Grapevine roditis leaf discoloration-associated virus
HaIV	Helicoverpa armigera iflavirus
HaSNPV	Helicoverpa armigera single nucleopolyhedrovirus
HeIV	Heliconius erato iflavirus
OpMNPV	Orgyia pseudotsugata multiple nucleopolyhedrovirus
OsEV	Oryza sativa endornavirus
PnPV	Perida nuda picorna-like virus
SeIV	Spodoptera exigua iflavirus
SfRV	Spodoptera frugiperda Rhabdovirus
TaBV	Taro bacilliform virus
TnTED	Trichoplusia ni Transposable Element D virus
WSSV	White Spot Syndrome Virus

Supplementary Table S5 - Accession number and origin of SRA and TSA archives used for genome assembly. RPM values indicate the prevalence of BmLV derived reads per million total reads.

Project:	SRA:	Lab/Institute	Country	Year	Cells	RPM
**sRNA archives:**						
PRJDB5144	DRR068690	GALS, U Tokyo	Japan	2018	*Bm*VF	1328
"	DRR068691	"	Japan	2018	*Bm*VF	2829
PRJDB10429	DRR241076	GALS, U Tokyo	Japan	2020	*Bm*N4	2252
PRJDB2257	DRR006265	Ins. Gen, U Tokyo	Japan	2014	*Bm*N4	1796
PRJDB4053	DRR039989	IMCB, U Tokyo	Japan	2016	*Bm*N4	1083
PRJDB5376	DRR079253	Ins. Gen, U Tokyo	Japan	2018	*Bm*N4	1277
PRJNA419912	SRR6324419-24	Siomi, U Tokyo	Japan	2017	*Bm*N4	2295
PRJNA632342	SRR11781560+61	Siomi, U Tokyo	Japan	2020	*Bm*N4	5043
PRJNA383127	SRR5458683	Comp Med Center, Thomas Jefferson U	USA	2017	*Bm*N4	7430
PRJDB2929	DRR023337+40	Biomed. C.S. Med. Cen.	USA	2014	*Bm*N4	6889
PRJNA239665	SRR1333837+38	Mol. Bio., U Geneva	Switzerland	2014	*Bm*N4	7687
PRJNA789788	SRR17258733	Mol. Dev. Phys., KU Leuven	Belgium	2021	*Bm*N4	5956
"	SRR17258732	Mol. Dev. Phys., KU Leuven	Belgium	2021	Hi5	574
"	SRR24542361	Mol. Dev. Phys., KU Leuven	Belgium	2021	Hi5	4529
**TSA archives:**						
PRJDB3935	DRR035925	Ins. Gen, U Tokyo	Japan	2018	*Bm*VF	
PRJNA554660	SRR9685281	IMB Mainz	Germany	2019	*Bm*N4	

Supplementary Table S6 – Sanger sequencing results

IOZCAS-*Ha*-I *argonaute 2* - partial transcript
CCCGTCGACCTGGGTTCTGGATATGAAATGTGGACGGGACTGTTTCAGTCTGCCATTTTCACAAACAAATCGTTTATTAATATCGATGTGGCCCACAAAGGTTTTCCTAAAGCTCAGAGTATGATTGATTGTCTTGTAAAAGACTTCAACTTGGACCCTTATCGTCCTA<br>TAGATAACCAGAGGGGAGGAGACAATTTCGCAACATTTGTCAAGGGGCTCAAGGTGGTAGCCAGTTTAGTTGGTAACACGGCGACGGCCGGACATAAACGCGAGTTCGTATGTAACGGGGTAGTTGGACCTCCGGACAAGCTCACATTCCCTATCACTGAGTCGGA<br>TGGCCGCTCTCGCAAGCTCACTGTCGCTGAATACTTTGCCAAGGAAAAGCAATATAGACTGAAATATCCGCATCTGAACTGCTTGTGGGTGGGTTCCCGTGAAAGATGCATTTACTTCCCAATGGAACTCCTCAATGTTTCCTATGGACAGCCTTTAGCAAGACAACT<br>AAATGAAATGCAAGTCTCAAAAATGGTGAAGGAAGCGGCAACACCGCCTGATGAGCGTTTGGCTAAAATTAAAGAAGTTATTTCAAATATGCAGTACTCCCAGAATAAAGATTTCAAGCAGTTTGGACTGGAGATTTCTGACAAATTCTACACGGTCCAGGCCAAA<br>ATTTAGATCCACCCGTTTTGGAAGTTGGAAATGCAAAAGTTACTCCCAGGAAGGGCCAATGGCAGGCTAATCGCCTCTTGAAGGCGGAAGCACTCCAGTCATGGGGTCTCATCGCCGTCGATACAGACCCGAGAATCGATTATGACAGCATGATTTCACTGATTAT<br>CAGCACCGGCAATCAGTTGGGCATGAATGTTTCCAAACCAAAGTTCGTTAATCTTGGCGCTAGAATGACGTCACTCCACGGCATTCTGATGAATGCTTACAAAGATGTCAGATTCGTTTTCGTGATCGTCTCTGCTAGGGGACGGGATGATTATCACAAGGTAAAGCA<br>AATGGCCGAACGTGAAGTTGGCATTCTGACACAATGCATTCGAGAGATGACTGCGCGTCGTATAAACCAGATGACAGCCAAGAACATCTTACTCAAGGTGAACTCCAAACTGATGGGTGTTAACCAAGCGATTGAACACAACACATTGCCAAAGTGTTTGAGGGAC<br>GGCAATGTCATGATTGTCGGCGCTGACGTCACTCATCCATCGCCAGATCAGTCAAATGTTCCCAGTATTGCAGCGGTAACGGCGTCCATCGATCCGCGATGCTATATGTATAACATCGAACTCAGCGTGCAGACACCTAAGAAAGAGATGATTGTGGAGTTCGAAGA<br>TATGATGTTCGATCACCTCAAGGTGTACAAGGATCGCAACAACTCCCTGCCAAAGAAGA
Bombyx mori latent virus (extracted from Hi5 cells)
GAATGGCTTTCACCAATTTAGTCGACACTCTCGCCAACACCATCCACCGAGACGCCATAACGGCTCCTCTAGTGGAAACGGCCATCAGCAATTTCCGTCACAAGCTTCAACTTTACCCATATCAGGTAAATTCCAAGCTCATCCCATTGCTCAATCAACTCGGAATAGGAGTCACCTCCTATGGCACTTCACCTCATCCTCA<br>TGCCGCTCATAAGGCCATAGAAACTTACCTCCTGTTTGAACATTGGAATCATCTTGCCAGAGTCCCTTCTACGGTAATGTATATGAAACCTGAAAAATTTCAAAAACTTCAGCAATTAAACCCAAACTTTGCTTCCCTCATTAACTTCAGGCACACCCCAAAGGACATTACTCGGTACCCCGTTAGCAACCCTCATCCTGTC<br>GAAACTGAAGTAGCGTTCATGCACGACGCTCTCATGTTCATCACCCCTTCCCAAATTTTAGGCCTTTTCAAGGACTCCCCTTCCATGACCTCACTCTATTGTTCTCTTATAGTCCCTGCCGAAGCCGCTTATGGAGTGCCTTCACTCTTCCCTGATCTCTACAGCTATACAATTAAAGATGATCAGCTGGTGTACTCTCTCGAA<br>GGAAATGCTACCGGCAATTACACTCAACCTCTCTGTTCTTTGGATTGGCTCCGCCGCTCAGGTATTTCATCAGGCGACCTTCATCTATCTGTCACTTTACTTGAATCCTTCGTTTCCGTCCATTCCATTCTAATCACTCGAGTTCCTCAACCTCCCCTCCTGGCGAACATGTATTCTTAACTCCTCCAGCTTCCCTGCTTCCTA<br>ATCCAGAAGGATTGGAACTGCCGATCAAATCACGACTCGTGCCAACTGAAGTTTACAATTCTTTGTTTACTTATGTTCGAGCCGTTCGCACGCTTCGCGTCACTGATCCTTCCGGATTCGTTAGGACGCAACGTCAAAAACCAGAACATTCATGGGTACAATCCTCAGCCTGGGATAATCTCGCCAACTTTGCCCTATTGAC<br>GTGCTCGGCCCGCCCTTCCTTAGAGTATGGTTTCTGCTATAACTCATACAAGCTTTTATCACTCTGGATCGTGCGTACATTGCGTTCGATATCAGCTTATCACACTGGTTCTCTTCTCACCATCCCAATTCTTCACCATCTTTCCCCTTATCAGCTGTGTTATCGAACTCATACCTTTAGATGGCTGCCCACTCATTTGGACTTCC<br>ACAAAACCTTGCCCTCTTTGATTCACAGAGCCTTCGCTAGCATTACTGGCCGCTACCTCACCAACTCACCAGAGTTCCCCACTTCAATCTTTTCTATTAATCATTTATTACAGCCTTTTGGCAAGTCATTGCAAAAATTTCCTCACCATTCCTTAGTCATTCGACCAAATCCTGCCTCCTTCATTGTTCGCTGTTCCACTCTTCT<br>ACAACGAAGGTGGCTCTTGCTTGGTTTGTCTGCCCTCACTGGCATTTGGGCCTATTACCATCTCCGCCGTGGCAACTCACCTCAAGAAAAGTCCGATGCTTATCTCTCTTATTTCCACCCGGATCCCTGGAGACTCACCATTCGAACATCTAATGTGATGGCTATTCCTCATTCTTTCTTCCCCGATTCAGGCAACTCGACTCC<br>TTCACCGTCTACACAACCGTCCCCTGACCCCATTGTTATCGAACAACCCTCGTCTAACGTCTCAAAACGTCATCTGGTTCCAAACCAAGCGTACCCTACACCGGCTCATCCTGAGACACCCCCAAGCCCCAGTAAACAAGAGATAAAATCCGGTGAGACTCTTGCCACCGGCTTTCCAAATGTTGCGACCCTGAGCGGCCCC<br>TCGGAAGCCTCGAAACCAGACGCCCCTCCTACTCCTTTAAGTTCCGATCCAACTGCTCATGGGCCCATCCTCCCTCATAAAGAACTTTTTGGCATTTCATCTCCCGATCACGAGTGCACCTTTTTAAATCGTAAGCGACTCAATGTTTCAACGCTTCCTTTCCCGGATCAGCCCTGTCTTCTTGTTGCTTTTGCTCAAGCCTCA<br>AATTCTACCCCTCAGCAAGTATGGGCTCACCTTTGCACCCTTTTCCCTGATTCCCTTCTCGATGGTCCCTTAGAACGCTCTCAAGGCTTCAGCTCAGAACACCTCGAAGCTCTCGCCTGGAGTTTGAATTATCACGTCTCTTATCGCCATGGCGAACATGTCAACACCATAGGCCCCGATGACGCACCACTGCTCCGCCTTAT<br>TTACACTGGTGATGGATCCATAGGTCATTGGGCTGCCGACGAATCCCCTCTCTCCCCCCCCCTTTCCTCTATTCGAGGCTCGGCAAAAACCCTCAATTCATTTGCTTCAACAGCTATTCGGTTTAGAGACTCTCACGGCAATCTCCTACCATTTCGACAAGTTCATGAGTACAAGCTCTGTAAGCCCAGGGCAAAAAATTTGG<br>CCTCAAACATGAAAAATGAGACTGATGGAGTTATACAATCTAGCCTCCGAGCGGCATCATCAGACCCCACTTTTTTCCAACGCCTTGACCAAAGAGCCGACTTCGCCCCACAAGTTTCCGTGCAGCTCATTCACATTACTGGATTTCCTGGTTGTGGGAAAACTTTCCCTGTCACTCAGCTCCTCAAAACCAAAGCATTTAA<br>AAGCCAGTACAGAGTCGCCGTCCCCACCACCGAGTTGCGTACCGAATGGAAAGACCACATGAAACTCCCTGCTAGCGACGTTTGGCGAGTCTCAACCTGGGAAACCTCTCTCATGAAATCAGCACCCGTCTTGGTTATCGACGAAGTGTACAAAATGCCTCGAGGTTTCCTGGATCTAGCTCTCCTAGCTGATCCAGCCAT<br>CCAATTTGTCATCCTTCTCGGAGACCCATGCCAAACCGTCTATTCTTCCGTCAATCCGGACTCTTCTAATTATCGTCTGATTTCCGAAGTCGAACATCTTAAACCGTATCGTGATTTTTACTGTCTCTGGACCCACCGACTTCCTCTTCGCCTAGCTCGATTCTTCGGCGTTTCGACTACGAACCCTCAAGAAGGTTTTATCGGA<br>CGTCGAGACAATCCACACAAATCTTATCCCATATTAACATCAAGTCAGCAGACGGCGCGAGTCTGTGCCGGTACAGGCCATCGAGCGCTCACTTTTTGCTCCTCTCAAGGTTCAACTTTCCAGGCTCCTGCCCAAATCTTCATCGACTCCAATGTGGCTTCTGTCCACGTTTCTGCTACTCTCGTCGCCACAACTCGTTCCCG<br>TTCTGGAGTCATTTTTACGGGAAACCATCGATTGTTCCAATCAAGACCTGGTACGGCGCCACTCTTCGAGGCTATGCTCAATGACCAACCCTTCAACTTCCTCAACACATTTGCTAATGAACTTGGAGGCATGGAACTCATTACATCACCGATCAAAGAACGAAAGACCATTCTTCGTGGCGGGTCTTATGACTTTTCAGCT<br>TTCCGAAGCCACCAAAAAAGACATCACCGAATGACCAACTTTAAATTCGATCCAGCTCATATCAGGGCTCCTCGCCCTGCATCTGCCAAACCACTACCTGCTAATGACACCTCTGACATAATAATTTTTGCACCTGAACTTTTTGACCTCCAGTCTTCAGCCATCCCGAGACTTGACACTCATCATATTCCAGAAACTCGTCG<br>TCCTCTCCATTTCGATATTCCTTCTTCTCTCCCTACCAAAGTGGACATTAGTAGTGTTGAATCTACAGACACCGCCATAGAACCTGTTTATCCCGGCTGCGACTACAAAACTGTGGCAGCTCTAATGATGGATCCCCGGGACCCCGACTCCCTTGAAATCCGCCACAAGGGGGAGTTCTCCAATCAATTTCCATGGGTCGATC<br>TCCCTTATGAGAATGGCGCCCAAACCCTCAGCGTTATAGCACCCAAACATGACTCGAAAAATGACCCGACTTTACTTTCGGCCTCCATAGCCAAAAGACTCCGTTTTCGACCCTCCTTCCTTCCTTACAGACTTTCTCCTTCTGACGAAGTCCTTGGCACATTTCTGTTTTCATCTCTATGTCGAGCCTATAAACGACATCCCA<br>ATCATAAAGTTCCTTTTCAGCCCGACCTTTTCGTTGAATGTATCAATCTTAATGAGTACTCACAGCTTTCCAACAAGACTCAAGCTGTCATCCAAGCGAACGCAAATCGTTCGGATCCCGACTGGCGGTACACTGCTGTCCGCATATTTTCAAAAACTCAGCATAAAATCAACGAAGGCTCAATTTTCGGCCCTTGGAAAGC<br>ATGCCAGACATTAGCCTTGATGCATGATGCCATTGTCTTAATTTTTGGCCCGATCAAGAAATATCAGCGAATTTTCGACAGTAAAGATCGTCCTCCCAACTTGTTTGTGTATGGAGGCCAAACTCCATACGATCTCTCTCGGTTTGCTTCGTCCCATCTGAAACCATTACAAATTCATGTCTGCAATGACTATACTGCGTTTG<br>ACCAATCTCAGCATGGTGAGGCCGTTGTTTTGGAACGGAAGAAAATGGAGCGGCTTTCCATTCCATCTCATCTTATAGACCTCCATTGCTACATCAAAACCAACATTACAAGTCAATTTGGACCTCTGACCTGCATGCGCCTCACCGGCGAGCCCGGCACTTATGACGACAACACGGATTACAATATTGCCGTCATTTACAG<br>CGAGTACGACATCACCACTCAAACTGTCTTTGTCTCAGGTGATGATTCTTTAGTATCACCTCCACCTCCTGCTCACCCCGATTGGGACGCCGTTTCTCCACTCCTTTCTCTCACATTCAAAAAAGAGTTTGTCAGAAACGGTCTCTTCTGCGGCTATTACTTGGGACCGGCCGGAGCCATTCGTGCTCCCCGTGCCTTACTCGC<br>TAAACTTGTGTTAGCCCTAGACGACGGAACCCTTTCTGACAAAATAGCGTCTTACCTCAGTGAGTTTGTTGTAGGCCACTCTCTCGGCGACGCTTTCTGGACTCTCTTGCCAGCAGATCAAGTAATTTACCAGAGCGCGCTTTTTGATTTCTTTTGCCGTGAATGTTCTAAGGAACAAAAACTCGCCCTCAAAATAGGGGAAG<br>TGCCCGAACATCTTTGTCAGAAAATGCTTTCCCTCGGTTTCTCCTGGTTATCACACCCCCTTTATGCTCTTCTGGATAGGACCGCCCGCCTACGTTTATTAGCCCGCACTCGTTTCGCCCCCCTCTTTTCCGATCCTTCTGTAGAGGGTGTTTTGCAACTAGATTTCTGATGACCTATTCATGGAAGAAATTGTGCCTTTAGTCG<br>CTTCTGCCGCTTCGGCCATTCCGTCCTTGGTCAATGCTTTCTCTTCTTCCAAACCCGCTCAAACTGATCACCCGTCTGCGCGTTCCATGGATATGCAACCTTTGCCTAGCTCTGACAGCGTTGTTCTGTCTAGCCAACCGCTCCCGCCGGCTCCTCCGCCCCCGCTCGGATCTTCCCTGGGACGATCTCCTCAACGTTTGATCAT<br>CCCTTTTCAACGCCTGTACTTCGATCTTACTGGCACCGAAACCAAATCGAACAGTGTCACTGTGCAATCACTACCGAACGTCTCCTCCATCATCAAAGGCTACCGCGACGCGTATCTTGTCAATCTTGAAGCTGTCGTTTTTCCTTCCGCCCCTAGCCTCAAAATACCAGTCACAGTTGACCTTTGTTGGACTACTGCTGACG<br>TCACAGTTGAAGGAGTCAATGTGTTGGCCACCCCTTCATCCGCTCGCATTACTATTGGCGGTCTCGCCCTTATGCATCAAGCTACCCTCCCCTGCGATCTCGGCTACATCAACCCGATCATCAAATCCCCGATTCCATACACCAACCACCCCAGACTTAACATCCATTTCCATCAATCCCCTGACGCCGTACTAGAAGGAGTT<br>CGCGCCGGGGTTAAAGCCTCCGTTGTCATCAGAGGCTCTATCAGCGTATCTCACCCCCTCGTCACTGGGCATGGCTGAAGGGGTTTTGCTCCCTTATTTTCAAGTGAATGATGTATCACAAGCTTCGCGCCTTATTAGTCGTCATTCCACGCATACTTCGCTCCCCTCCTCCAACTCACCCCCTCATTGAAGACGTCGTTATGG<br>CCACAAACCAAGCCATCATCGACTATAAAGTCAAAATCGCCGATAATGATTTAGTAACTCATAAGGAACTGGCCCTCAAGGTTTCTTCTATCATAGGCACCCGAGTCTACGTCTTCGATCCCTCTTGCTACTTTTAACTCCTCCCTTCGATACCGTATTGTACGACAACATCATGACGGTTCGTAAGAACAACAAAACTGC<br>TCTTCTCTCTGCATCTATTCAAGCTTCTCTCCCTTCCTCCGAGATCTACCGCCAGTTGATAGATCCTCGCCATGTTTCTTCCGACTCCTTTGGAGTTTACGTAAAATCTTAATCCCTTATTCTTCACAGTTTTGGGTTGTTAGTTTCATCCCGCTCT

Supplementary Table S7 – Amino acid difference matrix of the replicase polyprotein between newly assembled and reference BmLV genomes. Cells are shaded from high (dark) to low (light) percentage difference. The reference BmLV genome and the genome of the BmLV variant employed in this research are written in bold.

% Different AAs	DRR068691	SRR6324419-24	SRR11781560-1	SRR24542361	**SRR17258732 (this paper)**	KJ433990	AB186123.1	**AB624361.1 (reference)**	DRR241076	DRR035925	DRR068690	SRR1333837-38	SRR17258733	DRR079253	DRR039989	DRR023337+40	SRR5458683	SRR9685281	DRR006265
DRR068691	0	3.15	3.03	3.32	3.66	2.17	2.46	3.66	3.49	3.32	3.2	2.35	2.35	2.35	2.46	2.46	2.4	2.4	2.4
SRR6324419-24	3.15	0	1.26	4.41	4.75	3.78	3.43	4.98	4.86	4.69	4.58	3.49	3.43	3.43	3.55	3.55	3.49	3.49	3.49
SRR11781560-1	3.03	1.26	0	4.41	4.75	3.83	3.49	4.86	4.75	4.58	4.46	3.55	3.49	3.49	3.49	3.6	3.55	3.55	3.55
SRR24542361	3.32	4.41	4.41	0	0.69	2.75	2.75	4.29	4.23	3.89	3.89	2.69	2.63	2.63	2.75	2.75	2.69	2.69	2.69
**SRR17258732 (this paper)**	3.66	4.75	4.75	0.69	0	3.15	3.32	4.69	4.63	4.29	4.29	3.2	3.15	3.15	3.26	3.26	3.2	3.2	3.2
KJ433990	2.17	3.78	3.83	2.75	3.15	0	1.43	2.86	2.97	2.57	2.46	1.66	1.6	1.6	1.83	1.72	1.66	1.66	1.66
AB186123.1	2.46	3.43	3.49	2.75	3.32	1.43	0	2.8	2.86	2.52	2.4	1.77	1.72	1.72	1.89	1.83	1.77	1.77	1.77
**AB624361.1 (reference)**	3.66	4.98	4.86	4.29	4.69	2.86	2.8	0	2.69	2.12	2	3.26	3.2	3.21	3.26	3.32	3.26	3.26	3.26
DRR241076	3.49	4.86	4.75	4.23	4.63	2.97	2.86	2.69	0	1.6	1.49	2.97	2.92	2.92	2.92	3.03	2.97	2.97	2.97
DRR035925	3.32	4.69	4.58	3.89	4.29	2.57	2.52	2.12	1.6	0	0.11	2.92	2.86	2.86	2.92	2.97	2.92	2.92	2.92
DRR068690	3.2	4.58	4.46	3.89	4.29	2.46	2.4	2	1.49	0.11	0	2.8	2.75	2.75	2.8	2.86	2.8	2.8	2.8
SRR1333837-38	2.35	3.49	3.55	2.69	3.2	1.66	1.77	3.26	2.97	2.92	2.8	0	0.06	0.06	0.29	0.17	0.11	0.11	0.11
SRR17258733	2.35	3.43	3.49	2.63	3.15	1.6	1.72	3.2	2.92	2.86	2.75	0.06	0	0	0.23	0.11	0.06	0.06	0.06
DRR079253	2.35	3.43	3.49	2.63	3.15	1.6	1.72	3.21	2.92	2.86	2.75	0.06	0	0	0.23	0.11	0.06	0.06	0.06
DRR039989	2.46	3.55	3.49	2.75	3.26	1.83	1.89	3.26	2.92	2.92	2.8	0.29	0.23	0.23	0	0.23	0.17	0.17	0.17
DRR023337+40	2.46	3.55	3.6	2.75	3.26	1.72	1.83	3.32	3.03	2.97	2.86	0.17	0.11	0.11	0.23	0	0.06	0.06	0.06
SRR5458683	2.4	3.49	3.55	2.69	3.2	1.66	1.77	3.26	2.97	2.92	2.8	0.11	0.06	0.06	0.17	0.06	0	0	0
SRR9685281	2.4	3.49	3.55	2.69	3.2	1.66	1.77	3.26	2.97	2.92	2.8	0.11	0.06	0.06	0.17	0.06	0	0	0
DRR006265	2.4	3.49	3.55	2.69	3.2	1.66	1.77	3.26	2.97	2.92	2.8	0.11	0.06	0.06	0.17	0.06	0	0	0

Supplementary Table S8 – Raw Ct values for amplicons of housekeeping genes or viral genes in infected IOZCAS-*Ha*-I cells.

**Ct values (average of 2 wells) for HKG and viral amplicons of infected samples**					
Timepoint	Replicate	*α-Tub*	*RPS18*	*rep*	*cp*
Day 4	1	16.294	21.857	11.743	7.524
	2	16.321	21.935	11.976	7.413
	3	16.125	21.724	11.903	7.627
	4	16.219	21.807	11.975	7.445
Day 5	1	16.090	21.917	11.604	7.614
	2	16.401	22.112	12.020	7.786
	3	16.061	21.778	11.454	7.370
	4	16.072	21.901	11.660	7.550

**Ct values (average of 2 wells) for HKGs of infected samples (replicate 1 of each timepoint)**			
Timepoint	*α-Tub*	*RPS18*	*18S*
T0	17.196	23.251	7.394
4h	17.622	23.022	7.574
Day 1	15.948	21.72	6.566
Day 2	15.44	21.534	6.196
Day 3	16.13	21.959	5.961
Day 4	16.414	21.991	6.098

Supplementary Table S9 - Number of subjects at risk during *H. armigera* infection experiment.

Day of event	Mock infected	BmLV infected
0	94	96
1	94	96
2	82	83
3	70	70
6	58	58
8		46
12		45
14	45	44
15	40	41
16	39	39
17	38	38
18	37	35
19	34	34
20	27	25
21	21	21
22	15	18
23	11	16
24	8	12
25	6	9
26		4
27	5	
28	3	2
29	2	1
30	1	

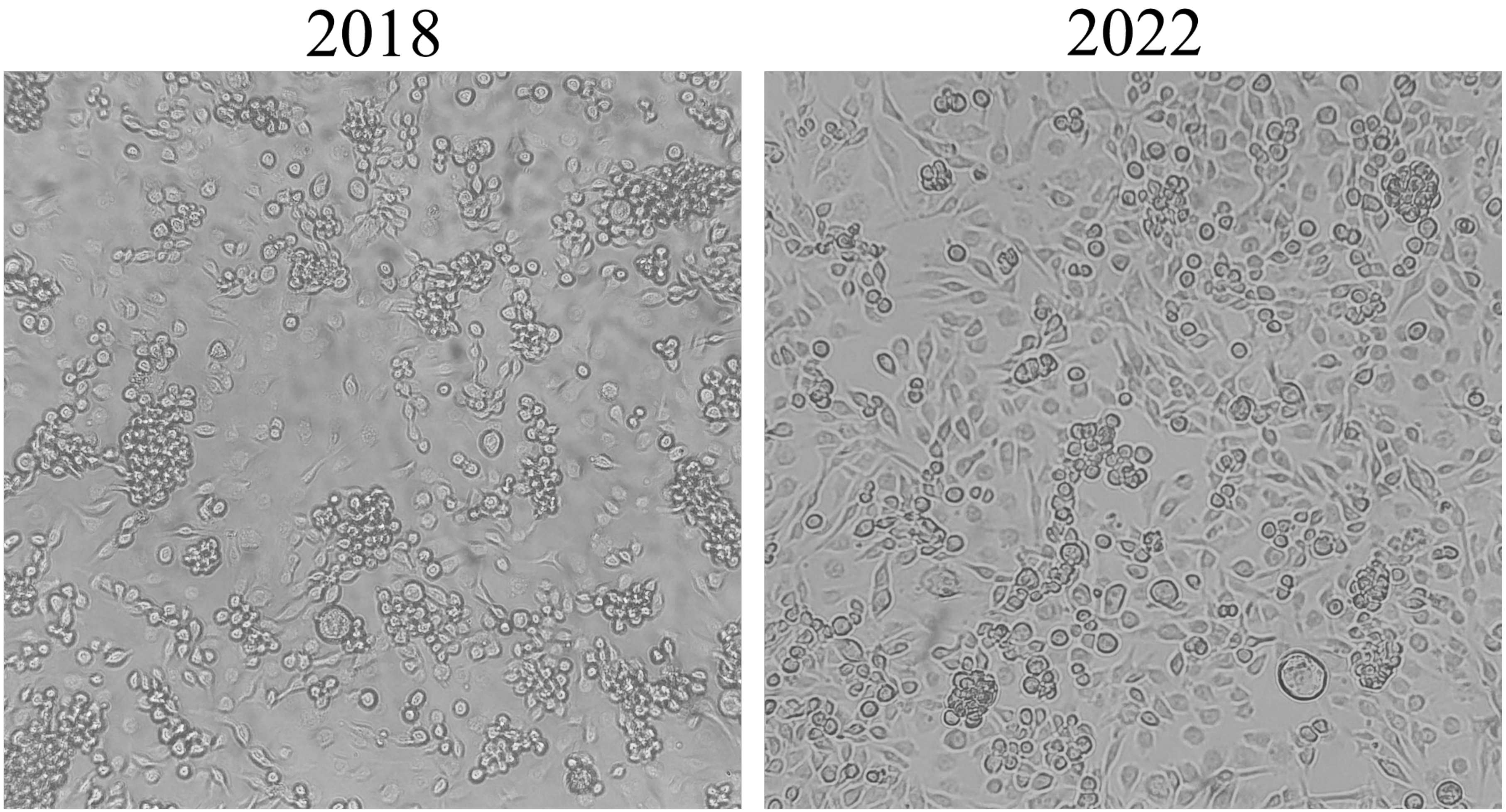

Supplementary Figure S1 - Photomicrograph (100x magnification) of IOZCAS-*Ha*-I cells upon arrival in our lab in May 2018 (left) and IOZCAS-*Ha*-I cells on day 4 after the mock infection used for this study in 2022 (right). The form of the cells varies from spherical to spindle-shaped.

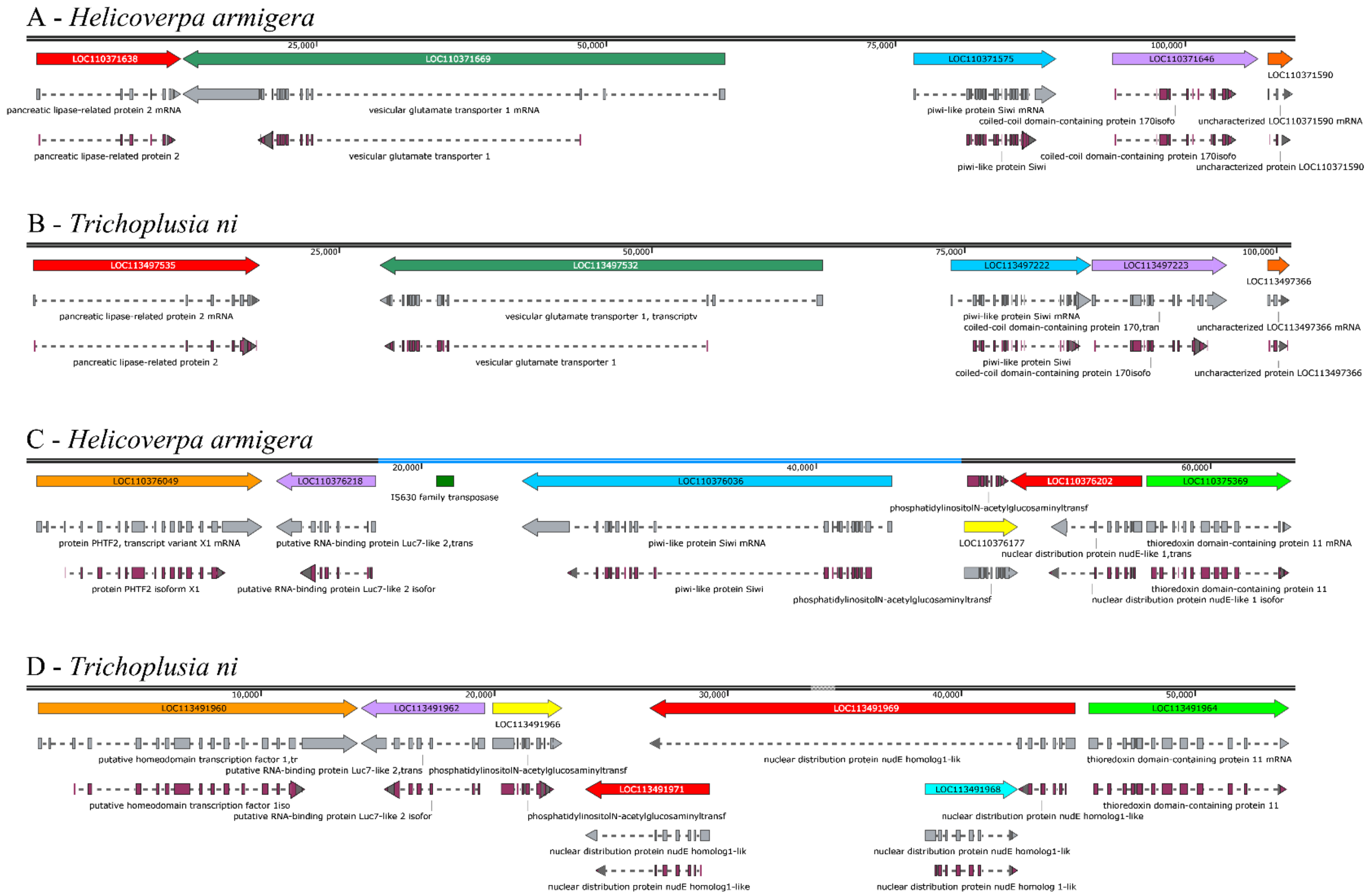

Supplementary Figure S2 - Genomic loci of two *siwi* genes (blue arrow) in *Helicoverpa armigera* (A) (Chromosome 12, NC_064787.1) and (C) (Chromosome 17, C_064792.1), with the corresponding genomic loci in *Trichoplusia ni* (B) (Chromosome 9, NC_039486.1) and (D)(Chromosome 3, NC_039480.1), respectively. For simplicity, only one transcript variant and protein isoform are represented for each gene. The presumptive *H. armigera* transposed DNA sequence of figure C is indicated in blue. Data sourced from the National Center for Biotechnology Information.

```
1    MSEPGRGRSRGRAGRGGDNAGPPPRKPGEQQQQRPPQQQGPPGPRPQPPTAYGAPAMGPPVRAGVPVAASPAGRASHRPTPTTHQEHPGDVDVQERMRSM  100
1    .................................................S.F....L.........TP.P......L.L.....................  100

101  QIS-TPQVGAGESASVIGRGSRRGGGRILPEQITILRTRPTTIQSKKGTSGTVIDLKANYFTVNTTPQWRLYQYHVDFSPEEDNTAVRKGLLRVHANTLG  199
101  ...AAS.......S.............V....M...........T.......................................................  200

200  GYLFDGAVLYTVKRLHPDPLELYSDRKTDGERMRILIKLTCDVSPGDYHYIQIFNIIIRKCFQLLNLQLMGRDYFDPVAKIDVPEYRLQIWPGYKTTINQ  299
201  ....................................................................................................  300

PAZ
300  YEDRLLMVTEITHKVLRLDNVLQMLSEYAATKGSNYKKIFLEDIVGKIVMTDYNKKTYRVDDVSWQASPKSTFKMRDENITYMEYYQKKYNIRIHDGGQP  399
301  ................................................................................S...................  400

PAZ
PIWI
400  LLISRSKPRDIRAGMPELVFLVPELCRQTGLTDDMRANFKLMRALDNHTKIGPDVRIQKLLKFNQRLTSCREVVKEMGDWSLTLSHELVSFKGRQLPPEN  499
401  ....................................................................................................  500

PIWI
500  IVQGNNAKYPAGDTTEGWTRDMRSKPLLDISQLPSWVVITPERQRRDAEGFIDLIMKTAGGVGFRMPKPEIIPIRNDGPMEYANACENAIARKNPAMILC  599
501  ...............................................S...................R................................  600
```

Supplementary Figure S3 – Sequence alignment (from AA 1 to 600) of *H. armigera* SIWI proteins XP_049696273.1 (upper row) and XP_049699728.1 (lower row). Conserved domains predicted with the NCBI conserved domain search tool. Dots represent identical AAs. No mutations are present in the C-terminal region omitted from the figure.

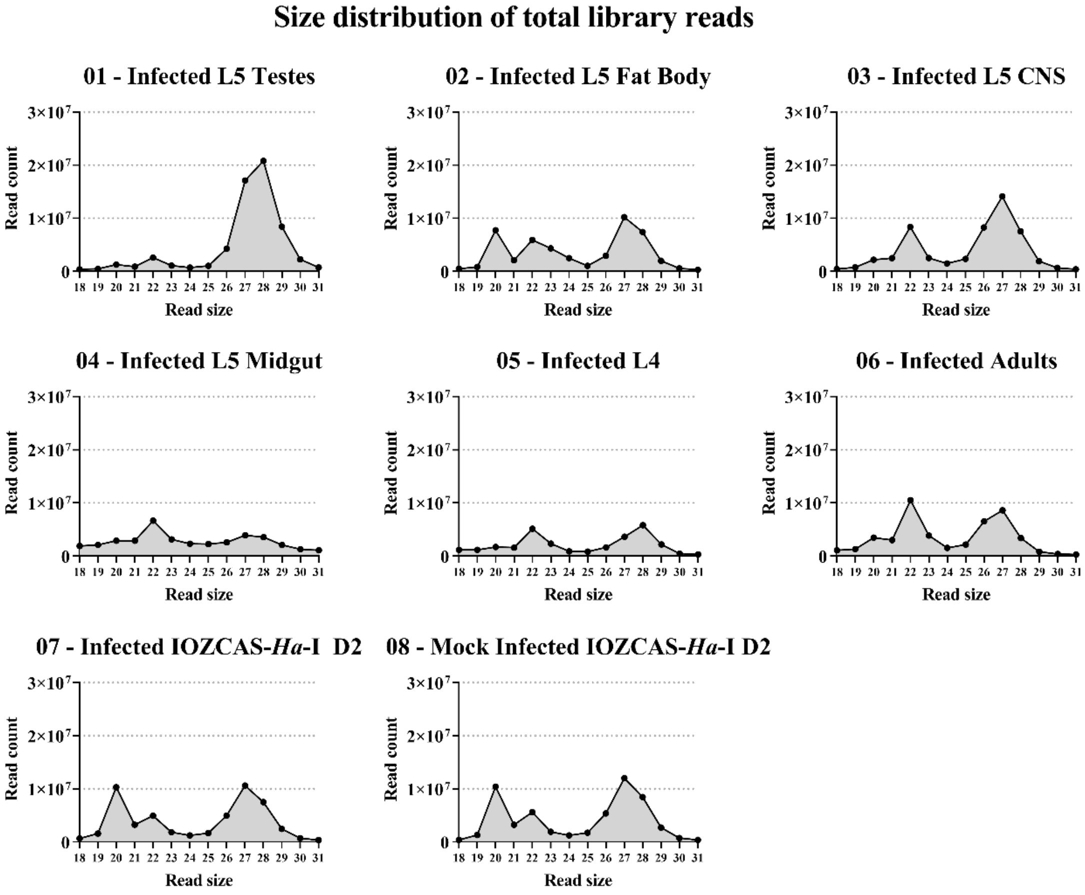

Supplementary Figure S4 – Size distribution of the total sRNA reads in the curated libraries for each sample.

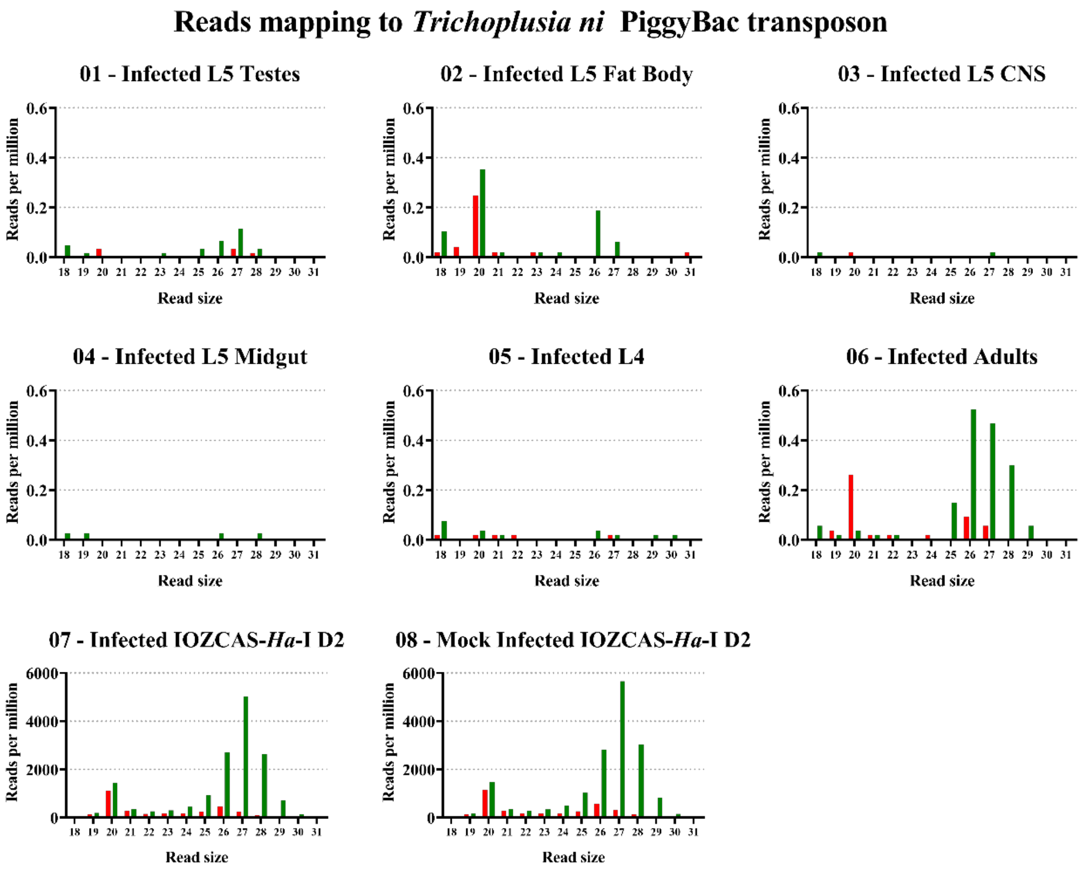

Supplementary Figure S5 - Analysis of sRNAs mapping onto the *Trichoplusia ni* PiggyBac transposon (GenBank: DQ340395.1) sampled from *H. armigera* animals (samples 01 to 06) and *Trichoplusia* cells (samples 07 and 08). Tissues were dissected on day 6 after infection, whole larvae were collected on day 2 after infection, whole adults were collected on day 1 of the adult stage, and the cells were collected on day 2 after (mock) infection. The frequency of sRNAs mapping onto the PiggyBac sequence is expressed in reads per million and divided by the read size (from 18 to 31 nt).

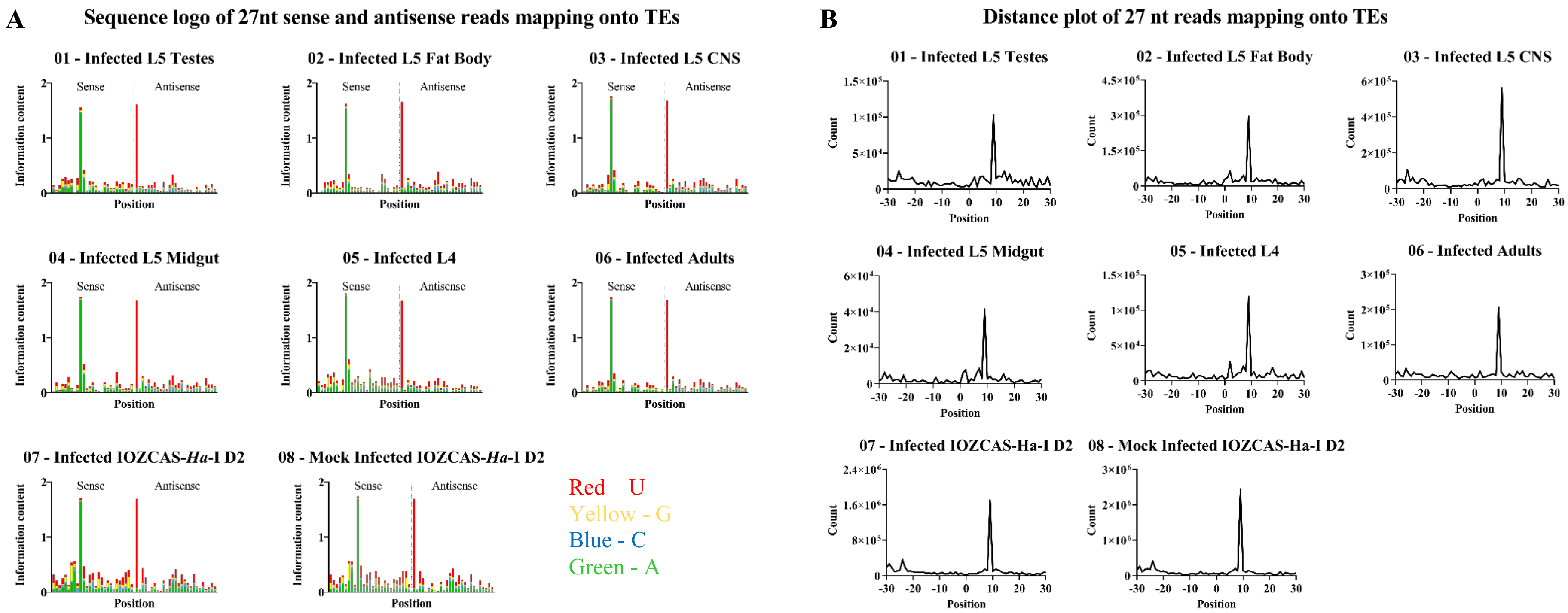

Supplementary Figure S6 – Secondary piRNA biogenesis pathway fingerprint analysis for reads mapping onto transposable elements. Tissues were dissected on day 6 after infection, whole larvae were collected on day 2 after infection, whole adults were collected on day 1 of the adult stage, and the cells were collected on day 2 after (mock) infection. (A) Nucleotide biases of 27 nt sized reads mapping onto transposable elements. Reads from *H. armigera* samples (01 till 06) were mapped onto the *Ha*RT1 sequence (GenBank: EU016079.1), reads from Trichoplusia samples (07 and 08) were mapped onto a PiggyBac transposon sequence (GenBank: DQ340395.1). For all samples, sense reads display a strong bias for an adenine on the 10th position whereas antisense reads have a strong bias towards an uracil on the 1st position. Information content (Y-axis) calculated according to the formula of Schneider and Stephens (1990). (B) Read distance plot of 27 nt reads mapping onto the same TEs. For all samples, a clear peak is seen at a distance of 9 nucleotides.

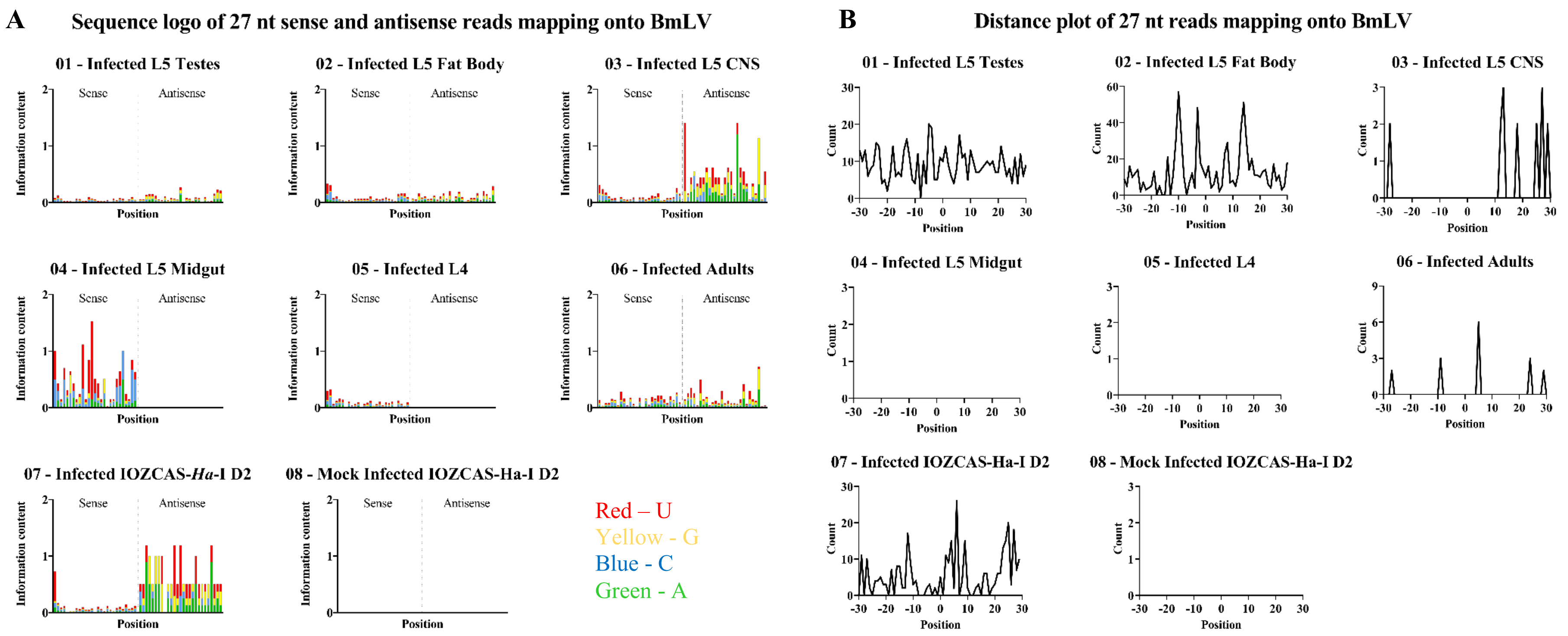

Supplementary Figure S7 - Secondary piRNA biogenesis pathway fingerprint analysis for reads mapping onto BmLV. Tissues were dissected on day 6 after infection, whole larvae were collected on day 2 after infection, whole adults were collected on day 1 of the adult stage, and the cells were collected on day 2 after (mock) infection. (A) Nucleotide biases of 27 nt sized reads mapping onto BmLV. No 1U or 10A nucleotide biases are found except for antisense reads from CNS where a 1U bias is present. Information content calculated according to the formula of Schneider and Stephens (1990). (B) Read distance plot of 27 nt reads mapping onto BmLV. No clear 9 nt distance peak is present in any sample.

Supplementary text S1 – Multiple sequence alignment of replicase polyproteins assembled from publicly available SRA datasets. Sequences aligned with MEGA7 (version 7.0.26) and then visualized with MView (version 1.63). Amino acids are colored according to their identity.

```
                          cov    pid     1 [      .       .       .       .       :       .       .       . 80
 1 DRR068690          100.0% 100.0%       MAFTNLVDTLANTIHRDAITAPLVETAINNFRHKLKLYPYQVNSKLIPLLNQLGIGVTSYGTSPHPHAAHKAIETHLLFE
 2 DRR068691          100.0%  96.8%       MAFTNLVDTLANTIHRDAITAPLVETAIRNFRHKLQLYPYQVNSKLIPLLNQLGIGVTSYGTSPHPHAAHKAIETYLLFE
 3 DRR006265          100.0%  97.2%       MAFTNLVDTLANTIHRDAITAPLVETAINNFRHKLQLYPYQVNSKLIPLLNQLGIGVTSYGTSPHPHAAHKAIETHLLFE
 4 DRR039989           99.9%  97.1%       MAFTNLVDTLANTIHRDAITAPLVETAINNFRHKLQLYPYQVNSKLIPLLNQLGIGVTSYGTSPHPHAAHKAIETHLLFE
 5 DRR079253           99.9%  97.2%       MAFTNLVDTLANTIHRDAITAPLVETAINNFRHKLQLYPYQVNSKLIPLLNQLGIGVTSYGTSPHPHAAHKAIETHLLFE
 6 SRR6324419-24      100.0%  95.4%       MAFTNLVDTLANTIHRDAITAPLVETAINNFRHKLQLYPYQVNSKLIPLLNQLGIGVTSYGTSPHPHAAHKAIETHLLFE
 7 SRR11781560-1      100.0%  95.5%       MAFTNLVDTLANTIHRDAITAPLVETAINNFRHKLQLYPYQVNSKLIPLLNQLGIGVTSYGTSPHPHAAHKAIETHLLFE
 8 SRR5458683         100.0%  97.2%       MAFTNLVDTLANTIHRDAITAPLVETAINNFRHKLQLYPYQVNSKLIPLLNQLGIGVTSYGTSPHPHAAHKAIETHLLFE
 9 DRR023337+40       100.0%  97.1%       MAFTNLVDTLANTIHRDAITAPLVETAINNFRHKLQLYPYQVNSKLIPLLNQLGIGVTSYGTSPHPHAAHKAIETHLLFE
10 SRR1333837-38      100.0%  97.2%       MAFTNLVDTLANTIHRDAITAPLVETAINNFRHKLQLYPYQVNSKLIPLLNQLGIGVTSYGTSPHPHAAHKAIETHLLFE
11 DRR241076          100.0%  98.5%       MAFTNLVDTLANTIHRDAITAPLVETAINNFRHKLQLYPYQVNSKLIPLLNQLGIGVTSYGTSPHPHAAHKAIETHLLFE
12 SRR17258733        100.0%  97.3%       MAFTNLVDTLANTIHRDAITAPLVETAINNFRHKLQLYPYQVNSKLIPLLNQLGIGVTSYGTSPHPHAAHKAIETHLLFE
13 SRR24542361        100.0%  96.1%       MAFTNLVDTLANTIHRDAITAPLVETAISNFRHKLQLYPYQVNSKLIPLLNQLGIGVTSYGTSPHPHAAHKAIETYLLFE
14 SRR17258732        100.0%  95.7%       MAFTNLVDTLANTIHRDAITAPLVETAISNFRHKLQLYPYQVNSKLIPLLNQLGIGVTSYGTSPHPHAAHKAIETYLLFE
15 SRR9685281         100.0%  97.2%       MAFTNLVDTLANTIHRDAITAPLVETAINNFRHKLQLYPYQVNSKLIPLLNQLGIGVTSYGTSPHPHAAHKAIETHLLFE
16 DRR035925          100.0%  99.9%       MAFTNLVDTLANTIHRDAITAPLVETAINNFRHKLKLYPYQVNSKLIPLLNQLGIGVTSYGTSPHPHAAHKAIETHLLFE
17 AB186123.1          99.9%  97.5%       MAFTNLVDTLANTIHRDAITAPLVETAISNFRHKLQLYPYQVNSKLIPLLNQLGIGVTSYGTSPHPHAAHKAIETHLLFE
18 KJ433990           100.0%  97.5%       MAFTNLVDTLANTIHRDAITAPLVETAISNFRHKLQLYPYQVNSKLIPLLNQLGIGVTSYGTSPHPHAAHKAIETHLLFE
19 AB624361.1         100.0%  97.3%       MAFTNLVDTLANIIHRDAITAPLVETAINNFRHKLKLYPYQVNSKLIPLLNQLGIGVTSYGTSPHPHAAHKAIETHLLFE
   consensus/100%                         MAFTNLVDTLANhIHRDAITAPLVETAIpNFRHKLpLYPYQVNSKLIPLLNQLGIGVTSYGTSPHPHAAHKAIETaLLFE
   consensus/90%                          MAFTNLVDTLANTIHRDAITAPLVETAIsNFRHKLpLYPYQVNSKLIPLLNQLGIGVTSYGTSPHPHAAHKAIETaLLFE
   consensus/80%                          MAFTNLVDTLANTIHRDAITAPLVETAIsNFRHKLQLYPYQVNSKLIPLLNQLGIGVTSYGTSPHPHAAHKAIETHLLFE
   consensus/70%                          MAFTNLVDTLANTIHRDAITAPLVETAINNFRHKLQLYPYQVNSKLIPLLNQLGIGVTSYGTSPHPHAAHKAIETHLLFE

                          cov    pid    81        .       1       .       .       .       .       :       . 160
 1 DRR068690          100.0% 100.0%       HWNHLARVPSTVMYMKPEKFQKLQQLNPNFASLINFRHTPKDITRYPVSNPHPVETEVAFMHDALMFITPSQILGLFKDS
 2 DRR068691          100.0%  96.8%       HWNHLARVPSTVLYMKPEKFQKLQQLNPNFASLINFRHTPKDITRYPVSNPHPVETEVAFMHDALMFITPSQILGLFKDS
 3 DRR006265          100.0%  97.2%       HWNYLARVPSTVMYMKPEKFQKLQQLNPNFASLINFRHTPKDITRYPVSNPHPVETEVAFMHDALMFITPSQILGLFKDS
 4 DRR039989           99.9%  97.1%       HWNYLARVPSTVMYMKPEKFQKLQQLNPNFASLINFRHTPKDITRYPVSNPHPVETEVAFMHDALMFITPSQILGLFKDS
 5 DRR079253           99.9%  97.2%       HWNYLARVPSTVMYMKPEKFQKLQQLNPNFASLINFRHTPKDITRYPVSNPHPVETEVAFMHDALMFITPSQILGLFKDS
 6 SRR6324419-24      100.0%  95.4%       HWNHLARVPSTVLYMKPEKFQKLQQLNPNFASLINFRHTPKDITRYPVSNPHPVETEVAFLHDALMFITPSQILGLFKDS
 7 SRR11781560-1      100.0%  95.5%       HWNHLARVPSTVLYMKPEKFQKLQQLNPNFASLINFRHTPKDITRYPVSNPHPVETEVAFLHDALMFITPSQILGLFKDS
 8 SRR5458683         100.0%  97.2%       HWNYLARVPSTVMYMKPEKFQKLQQLNPNFASLINFRHTPKDITRYPVSNPHPVETEVAFMHDALMFITPSQILGLFKDS
 9 DRR023337+40       100.0%  97.1%       HWNYLARVPSTVMYMKPEKFQKLQQLNPNFASLINFRHTPKDITRYPVSNPHPVETEVAFMHDALMFITPSQILGLFKDS
10 SRR1333837-38      100.0%  97.2%       HWNYLARVPSTVMYMKPEKFQKLQQLNPNFASLINFRHTPKDITRYPVSNPHPVETEVAFMHDALMFITPSQILGLFKDS
11 DRR241076          100.0%  98.5%       HWNHLARVPSTVMYMKPEKFQKLQQLNPNFASLINFRHTPKDITRYPVSNPHPVETEVAFMHDALMFITPSQILGLFKDS
12 SRR17258733        100.0%  97.3%       HWNYLARVPSTVMYMKPEKFQKLQQLNPNFASLINFRHTPKDITRYPVSNPHPVETEVAFMHDALMFITPSQILGLFKDS
13 SRR24542361        100.0%  96.1%       HWNHLARVPSTVMYMKPEKFQKLQQLNPNFASLINFRHTPKDITRYPVSNPHPVETEVAFMHDALMFITPSQILGLFKDS
14 SRR17258732        100.0%  95.7%       HWNHLARVPSTVMYMKPEKFQKLQQLNPNFASLINFRHTPKDITRYPVSNPHPVETEVAFMHDALMFITPSQILGLFKDS
```

```
15 SRR9685281       100.0%  97.2%       HWNYLARVPSTVMYMKPEKFQKLQQLNPNFASLINFRHTPKDITRYPVSNPHPVETEVAFMHDALMFITPSQILGLFKDS
16 DRR035925        100.0%  99.9%       HWNHLARVPSTVMYMKPEKFQKLQQLNPNFASLINFRHTPKDITRYPVSNPHPVETEVAFMHDALMFITPSQILGLFKDS
17 AB186123.1        99.9%  97.5%       HWNHLARVPSTVMYMKPEKFQKLQQLNPNFASLINFRHTPKDITRYPVSNPHPVETEVAFMHDALMFITPSQILGLFKDS
18 KJ433990         100.0%  97.5%       HWNHLARVPSTVMYMKPEKFQKLQQLNPNFASLINFRHTPKDITRYPVSNPHPVETEVAFMHDALMFITPSQILGLFRDS
19 AB624361.1       100.0%  97.3%       HWNHLARVPSTVMYMKPEKFQKLQQLNPNFASLINFRHTPKDITRYPVSNPHPVETEVAFVHDALMFITPSQILGLFKDS
   consensus/100%                       HWNaLARVPSTVhYMKPEKFQKLQQLNPNFASLINFRHTPKDITRYPVSNPHPVETEVAFhHDALMFITPSQILGLF+DS
   consensus/90%                        HWNaLARVPSTVhYMKPEKFQKLQQLNPNFASLINFRHTPKDITRYPVSNPHPVETEVAFhHDALMFITPSQILGLFKDS
   consensus/80%                        HWNaLARVPSTVMYMKPEKFQKLQQLNPNFASLINFRHTPKDITRYPVSNPHPVETEVAFMHDALMFITPSQILGLFKDS
   consensus/70%                        HWNaLARVPSTVMYMKPEKFQKLQQLNPNFASLINFRHTPKDITRYPVSNPHPVETEVAFMHDALMFITPSQILGLFKDS

                      cov    pid   161             .              .              .              2              .              .              .              . 240
 1 DRR068690        100.0% 100.0%       PSMTSLYCSLIVPAEAAYGVPSLFPDLYSYTIKDDQLVYTLEGNATGNYTQPLRSLDWLRRSGISSGDLHLSVTLLESFV
 2 DRR068691        100.0%  96.8%       PSMTSLYCSLIVPAEAAYGVPSLFPDLYSYTIKDDQLVYTLEGNATGNYTQPLRSLDWLRRSGISSGDLHLSVTLLESFV
 3 DRR006265        100.0%  97.2%       PSMTSLYCSLIVPAEAAYGVPSLFPDLYSYTIKDDQLVYSLEGNATGNYTQPLRSLDWLRRSGISSGDLHLSVTLLESFV
 4 DRR039989         99.9%  97.1%       PSMTSLYCSLIVPAEAAYGVPSLFPDLYSYTIKDDQLVYSLEGNATGNYTQPLRSLDWLRRSGISSGDLHLSVTLLESFV
 5 DRR079253         99.9%  97.2%       PSMTSLYCSLIVPAEAAYGVPSLFPDLYSYTIKDDQLVYSLEGNATGNYTQPLRSLDWLRRSGISSGDLHLSVTLLESFV
 6 SRR6324419-24    100.0%  95.4%       PSMTSLYCSLIVPAEAAYGVPSLFPDLYSYTIKDDQLVYTLEGNATGNYTQPLRSLDWLRRSGISSGDLHLSVTLLESFV
 7 SRR11781560-1    100.0%  95.5%       PSMTSLYCSLIVPAEAAYGVPSLFPDLYSYTIKDDQLVYTLEGNATGNYTQPLRSLDWLRRSGISSGDLHLSVTLLESFV
 8 SRR5458683       100.0%  97.2%       PSMTSLYCSLIVPAEAAYGVPSLFPDLYSYTIKDDQLVYSLEGNATGNYTQPLRSLDWLRRSGISSGDLHLSVTLLESFV
 9 DRR023337+40     100.0%  97.1%       PSMTSLYCSLIVPAEAAYGVPSLFPDLYSYTIKDDQLVYSLEGNATGNYTQPLRSLDWLRRSGISSGDLHLSVTLLESFV
10 SRR1333837-38    100.0%  97.2%       PSMTSLYCSLIVPAEAAYGVPSLFPDLYSYTIKDDQLVYSLEGNATGNYTQPLRSLDWLRRSGISSGDLHLSVTLLESFV
11 DRR241076        100.0%  98.5%       PSMTSLYCSLIVPAEAAYGVPSLFPDLYSYTIKDDQLVYTLEGNATGNYIQPLRSLDWLRRSGISSGDLHLSVTLLESFV
12 SRR17258733      100.0%  97.3%       PSMTSLYCSLIVPAEAAYGVPSLFPDLYSYTIKDDQLVYSLEGNATGNYTQPLRSLDWLRRSGISSGDLHLSVTLLESFV
13 SRR24542361      100.0%  96.1%       PSMTSLYCSLIVPAEAAYGVPSLFPDLYSYTIKDDQLVYSLEGNATGNYTQPLCSLDWLRRSGISSGDLHLSVTLLESFV
14 SRR17258732      100.0%  95.7%       PSMTSLYCSLIVPAEAAYGVPSLFPDLYSYTIKDDQLVYSLEGNATGNYTQPLCSLDWLRRSGISSGDLHLSVTLLESFV
15 SRR9685281       100.0%  97.2%       PSMTSLYCSLIVPAEAAYGVPSLFPDLYSYTIKDDQLVYSLEGNATGNYTQPLRSLDWLRRSGISSGDLHLSVTLLESFV
16 DRR035925        100.0%  99.9%       PSMTSLYCSLIVPAEAAYGVPSLFPDLYSYTIKDDQLVYTLEGNATGNYTQPLCSLDWLRRSGISSGDLHLSVTLLESFV
17 AB186123.1        99.9%  97.5%       PSMTSLYCSLIVPAEAAYGVPSLFPDLYSYTIKDDQLVYTLEGNATGNYTQPLRSLDWLRRSGISSGDLHLSVTLLESFV
18 KJ433990         100.0%  97.5%       PSMTSLYCSLIVPAEAAYGVPSLFPDLYSYTIKDDQLVYTLEGNATGNYTQPLRSLDWLRRSGISSGDLHLSVTLLESFV
19 AB624361.1       100.0%  97.3%       PSMTSLYCSLIVPAEAAYGVPSLFPDLYSYTIKDDQLVYTLEGNATGNYTQPLRSLDWLRRSGISSGDLHLSVTLLESFV
   consensus/100%                       PSMTSLYCSLIVPAEAAYGVPSLFPDLYSYTIKDDQLVYoLEGNATGNYhQPLpSLDWLRRSGISSGDLHLSVTLLESFV
   consensus/90%                        PSMTSLYCSLIVPAEAAYGVPSLFPDLYSYTIKDDQLVYoLEGNATGNYTQPLpSLDWLRRSGISSGDLHLSVTLLESFV
   consensus/80%                        PSMTSLYCSLIVPAEAAYGVPSLFPDLYSYTIKDDQLVYoLEGNATGNYTQPLRSLDWLRRSGISSGDLHLSVTLLESFV
   consensus/70%                        PSMTSLYCSLIVPAEAAYGVPSLFPDLYSYTIKDDQLVYoLEGNATGNYTQPLRSLDWLRRSGISSGDLHLSVTLLESFV

                      cov    pid   241             :              .              .              .              .              3              .              . 320
 1 DRR068690        100.0% 100.0%       SVHSLLITRVPQPPPSGEHVFLTPPASLLPNPEGLELPIKSRLVPTEVYNSLFTYVRAVRTLRVTDPSGFVRTQRQKPEH
 2 DRR068691        100.0%  96.8%       SVHSLLITRVPQPPPSGEHVFLTPPASLLPNPEGLELPIKSRLVPTEVYNSLFTYVRAVRTLRVTDPSGFVRTQRQKPEH
 3 DRR006265        100.0%  97.2%       SVHSLLITRVPQPPPSGEHVFLTPPASLLPNPEGLELPIKSRLVPTEVYDSLFTYVRAVRTLRVTDPSGFVRTQRQKPEH
 4 DRR039989         99.9%  97.1%       SVHSLLITRVPQPPPSGEHVFLTPPASLLPNPEGLELPIKSRLVPTEVYDSLFTYVRAVRTLRVTDPSGFVRTQRQKPEH
 5 DRR079253         99.9%  97.2%       SVHSLLITRVPQPPPSGEHVFLTPPASLLPNPEGLELPIKSRLVPTEVYDSLFTYVRAVRTLRVTDPSGFVRTQRQKPEH
 6 SRR6324419-24    100.0%  95.4%       SVHSLLITRVPQPPPSGEHVFLTPPASLLPNPEGLELPIKSRLVPTEVYNSLFTYVRAVRTLRVTDPSGFVRTQRQKPEH
 7 SRR11781560-1    100.0%  95.5%       SVHSLLITRVPQPPPSGEHVFLTPPASLLPNPEGLELPIKSRLVPTEVYNSLFTYVRAVRTLRVTDPSGFVRTQRQKPEH
 8 SRR5458683       100.0%  97.2%       SVHSLLITRVPQPPPSGEHVFLTPPASLLPNPEGLELPIKSRLVPTEVYDSLFTYVRAVRTLRVTDPSGFVRTQRQKPEH
```

```
 9 DRR023337+40    100.0%   97.1%      SVHSLLITRVPQPPPSGEHVFLTPPASLLPNPEGLELPIKSRLVPTEVYDSLFTYVRAVRTLRVTDPSGFVRTQRQKPEH
10 SRR1333837-38   100.0%   97.2%      SVHSLLITRVPQPPPSGEHVFLTPPASLLPNPEGLELPIKSRLVPTEVYDSLFTYVRAVRTLRVTDPSGFVRTQRQKPEH
11 DRR241076       100.0%   98.5%      SVHSLLITRVPQPPPSGEHVFLTPPASLLPNPEGLELPIKSRLVPTEVYNSLFTYVRAVRTLRVTDPSGFVRTQRQKPEH
12 SRR17258733     100.0%   97.3%      SVHSLLITRVPQPPPSGEHVFLTPPASLLPNPEGLELPIKSRLVPTEVYDSLFTYVRAVRTLRVTDPSGFVRTQRQKPEH
13 SRR24542361     100.0%   96.1%      SVHSFLITRVPQPPPPGEHVFLTPPASLLPNPEGLELPIKSRLVPTEVYNSLFTYVRAVRTLRVTDPSGFVRTQRQKPEH
14 SRR17258732     100.0%   95.7%      SVHSILITRVPQPPPPGEHVFLTPPASLLPNPEGLELPIKSRLVPTEVYNSLFTYVRAVRTLRVTDPSGFVRTQRQKPEH
15 SRR9685281      100.0%   97.2%      SVHSLLITRVPQPPPSGEHVFLTPPASLLPNPEGLELPIKSRLVPTEVYDSLFTYVRAVRTLRVTDPSGFVRTQRQKPEH
16 DRR035925       100.0%   99.9%      SVHSLLITRVPQPPPSGEHVFLTPPASLLPNPEGLELPIKSRLVPTEVYNSLFTYVRAVRTLRVTDPSGFVRTQRQKPEH
17 AB186123.1       99.9%   97.5%      SVHSLLITRVPQPPPSGEHVFLTPPASLLPNPEGLELPIKSRLVPTEVYNSLFTYVRAVRTLRVTDPSGFVRTQRQKPEH
18 KJ433990        100.0%   97.5%      SVHSLLITRVPQPPPSGEHVFLTPPASLLPNPEGLELPIKSRLVPTEVYNSLFTYVRAVRTLRVTDPSGFVRTQRQKPEH
19 AB624361.1      100.0%   97.3%      SVHSLLITRVPQPPPSGEHVFLTPPASLLPNPEGLELPIKSRLVPTEVYNSLFTYVRAVRTLRVTDPSGFVRTQRQKPEH
   consensus/100%                      SVHShLITRVPQPPPsGEHVFLTPPASLLPNPEGLELPIKSRLVPTEVYsSLFTYVRAVRTLRVTDPSGFVRTQRQKPEH
   consensus/90%                       SVHSlLITRVPQPPPsGEHVFLTPPASLLPNPEGLELPIKSRLVPTEVYsSLFTYVRAVRTLRVTDPSGFVRTQRQKPEH
   consensus/80%                       SVHSLLITRVPQPPPSGEHVFLTPPASLLPNPEGLELPIKSRLVPTEVYsSLFTYVRAVRTLRVTDPSGFVRTQRQKPEH
   consensus/70%                       SVHSLLITRVPQPPPSGEHVFLTPPASLLPNPEGLELPIKSRLVPTEVYsSLFTYVRAVRTLRVTDPSGFVRTQRQKPEH

                   cov     pid   321          .         .         :         .         .         .         .         4 400
 1 DRR068690       100.0% 100.0%      SWVQSSAWDNLANFALLTCSARPSLEYGFCYSSYKLLSLWIVRTLRSISAYHTGSLLTIPILHHLSPYQLCYRTHTFRWL
 2 DRR068691       100.0%  96.8%      SWVQSSAWDNLANFALLTCSARPSLEYGFCYSSYKLLSLWIMRTLRSISAYHTGSLLTIPILHHLSPYQLCYRTHTFRWL
 3 DRR006265       100.0%  97.2%      SWVQSSAWDNLANFALLTCSARPSLEYGFCYSSYKLLSLWIVRTLRSISAYHTGSLLTIPILHHLSPYQLCYRTHTFRWL
 4 DRR039989        99.9%  97.1%      SWVQSSAWDNLANFALLTCSARPSLEYGFCYSSYKLLSLWIVRTLRSISAYHTGSLLTIPILHHLSPYQLCYRTHTFRWL
 5 DRR079253        99.9%  97.2%      SWVQSSAWDNLANFALLTCSARPSLEYGFCYSSYKLLSLWIVRTLRSISAYHTGSLLTIPILHHLSPYQLCYRTHTFRWL
 6 SRR6324419-24   100.0%  95.4%      SWVQSSAWDNLANFALLTCSARPSLEYGFCYSSYKLLSLWIMRTLRSISAYHTGSLLTIPILHHLSPYQLCYRTHTFRWL
 7 SRR11781560-1   100.0%  95.5%      SWVQSSAWDNLANFALLTCSARPSLEYGFCYSSYKLLSLWIMRTLRSISAYHTGSLLTIPILHHLSPYQLCYRTHTFRWL
 8 SRR5458683      100.0%  97.2%      SWVQSSAWDNLANFALLTCSARPSLEYGFCYSSYKLLSLWIVRTLRSISAYHTGSLLTIPILHHLSPYQLCYRTHTFRWL
 9 DRR023337+40    100.0%  97.1%      SWVQSSAWDNLANFALLTCSARPSLEYGFCYSSYKLLSLWIVRTLRSISAYHTGSLLTIPILHHLSPYQLCYRTHTFRWL
10 SRR1333837-38   100.0%  97.2%      SWVQSSAWDNLANFALLTCSARPSLEYGFCYSSYKLLSLWIVRTLRSISAYHTGSLLTIPILHHLSPYQLCYRTHTFRWL
11 DRR241076       100.0%  98.5%      SWVQSSAWDNLANFALLTCSARPSLEYGFSYSSYKLLSLWIVRTLRSISAYHTGSLLTIPILHHLSPYQLCYRTHTFRWL
12 SRR17258733     100.0%  97.3%      SWVQSSAWDNLANFALLTCSARPSLEYGFCYSSYKLLSLWIVRTLRSISAYHTGSLLTIPILHHLSPYQLCYRTHTFRWL
13 SRR24542361     100.0%  96.1%      SWVQSSAWDNLANFALLTCSARPSLEYGFCYNSYKLLSLWIVRTLRSISAYHTGSLLTIPILHHLSPYQLCYRTHTFRWL
14 SRR17258732     100.0%  95.7%      SWVQSSAWDNLANFALLTCSARPSLEYGFCYNSYKLLSLWIVRTLRSISAYHTGSLLTIPILHHLSPYQLCYRTHTFRWL
15 SRR9685281      100.0%  97.2%      SWVQSSAWDNLANFALLTCSARPSLEYGFCYSSYKLLSLWIVRTLRSISAYHTGSLLTIPILHHLSPYQLCYRTHTFRWL
16 DRR035925       100.0%  99.9%      SWVQSSAWDNLANFALLTCSARPSLEYGFCYSSYKLLSLWIVRTLRSISAYHTGSLLTIPILHHLSPYQLCYRTHTFRWL
17 AB186123.1       99.9%  97.5%      SWVQSSAWDNLANFALLTCSARPSLEYGFCYSSYKLLSLWIVRTLRSISAYHTGSLLTIPILHHLSPYQLCYRTHTFRWL
18 KJ433990        100.0%  97.5%      SWVQSSAWDNLANFALLTCSARPSLEYGFCYSSYKLLSLWIVRTLRSISAYHTGSLLTIPILHHLSPYQLCYRTHTFRWL
19 AB624361.1      100.0%  97.3%      SWVQSSAWDNLANFALLTCSARPSLEYGFCYSSYKLLSLWIVRTLRSISAYHTGSLLTIPILHHLSPYQLCYRTHTFRWL
   consensus/100%                      SWVQSSAWDNLANFALLTCSARPSLEYGFsYsSYKLLSLWIhRTLRSISAYHTGSLLTIPILHHLSPYQLCYRTHTFRWL
   consensus/90%                       SWVQSSAWDNLANFALLTCSARPSLEYGFCYsSYKLLSLWIhRTLRSISAYHTGSLLTIPILHHLSPYQLCYRTHTFRWL
   consensus/80%                       SWVQSSAWDNLANFALLTCSARPSLEYGFCYSSYKLLSLWIVRTLRSISAYHTGSLLTIPILHHLSPYQLCYRTHTFRWL
   consensus/70%                       SWVQSSAWDNLANFALLTCSARPSLEYGFCYSSYKLLSLWIVRTLRSISAYHTGSLLTIPILHHLSPYQLCYRTHTFRWL

                   cov     pid   401          .         .         .         .         :         .         .         . 480
 1 DRR068690       100.0% 100.0%      PTHLDFHKTLPSLIHRAFASITGRYITNSPEFPTSTFSINHLLQPFGKSLQKFPCHSLVIQPNPTSFIVRCSTLLERRWL
 2 DRR068691       100.0%  96.8%      PTHLDFHKTLPSLIHRAFASITGRYLTNSPEFPTSTFSINHLLQPFGKSLQKFPHHSLVIRPNPASFIVRCSTLLQRRWL
```

```
 3 DRR006265       100.0%  97.2%      PTHLDFHKTLPSLIHRAFASITGRYLTNSPEFPTSTFSINHLLQPFGKSLQKFPHHSLVIRPNPASFIVRCSTLLQRKWL
 4 DRR039989        99.9%  97.1%      PTHLDFHKTLPSLIHRAFASITGRYLTNSPEFPTSTFSINHLLQPFGKSLQKFPHHSLVIRPNPASFIVRCSTLLQRKWL
 5 DRR079253        99.9%  97.2%      PTHLDFHKTLPSLIHRAFASITGRYLTNSPEFPTSTFSINHLLQPFGKSLQKFPHHSLVIRPNPASFIVRCSTLLQRKWL
 6 SRR6324419-24   100.0%  95.4%      PTHLDFHKTLPSLIHRAFASITGRYLTNSPEFPTSTFSINHLLQPFGKSSQKFPHHSLVIRPNPASFIVRCSTLLQRRWL
 7 SRR11781560-1   100.0%  95.5%      PTHLDFHKTLPSLIHRAFASITGCYLTNSPEFPTSTFSINHLLQPFGKSLQKFPHHSLVIRPNPASFIVRCSTLLQRRWL
 8 SRR5458683      100.0%  97.2%      PTHLDFHKTLPSLIHRAFASITGRYLTNSPEFPTSTFSINHLLQPFGKSLQKFPHHSLVIRPNPASFIVRCSTLLQRKWL
 9 DRR023337+40    100.0%  97.1%      PTHLDFHKTLPSLIHRAFASITGRYLTNSPEFPTSTFSINHLLQPFGKSLQKFPHHSLVIRPNPASFIVRCSTLLQRKWL
10 SRR1333837-38   100.0%  97.2%      PTHLDFHKTLPSLIHRAFASITGRYLTNSPEFPTSTFSINHLLQPFGKSLQKFPHHSLVIRPNPASFIVRCSTLLQRKWL
11 DRR241076       100.0%  98.5%      PTHLDFHKTLPSLIHRAFASITGRYVTNSPEFPTSTFSINHLLQPFGKSLQKFPCHSLVIQPNPTSFIVRCSTLLERKWL
12 SRR17258733     100.0%  97.3%      PTHLDFHKTLPSLIHRAFASITGRYLTNSPEFPTSTFSINHLLQPFGKSLQKFPHHSLVIRPNPASFIVRCSTLLQRKWL
13 SRR24542361     100.0%  96.1%      PTHLDFHKTLPSLIHRAFASITGRYLTNSPEFPTSTFSINHLLQPFGKSLQKFPHHSLVIRPNPASFIVRCSTLLQRRWL
14 SRR17258732     100.0%  95.7%      PTHLDFHKTLPSLIHRAFASITGRYLTNSPEFPTSIFSINHLLQPFGKSLQKFPHHSLVIRPNPASFIVRCSTLLQRRWL
15 SRR9685281      100.0%  97.2%      PTHLDFHKTLPSLIHRAFASITGRYLTNSPEFPTSTFSINHLLQPFGKSLQKFPHHSLVIRPNPASFIVRCSTLLQRKWL
16 DRR035925       100.0%  99.9%      PTHLDFHKTLPSLIHRAFASITGRYITNSPEFPTSTFSINHLLQPFGKSLQKFPCHSLVIQPNPTSFIVRCSTLLERRWL
17 AB186123.1       99.9%  97.5%      PTHLDFHKTLPSLIHRAFASITGRYITNSPEFPTSTFSINHLLQPFGKSLQKFPCHSLVIRPNPASFIVRCSTLLQRRWL
18 KJ433990        100.0%  97.5%      PTHLDFHKTLPSLIHRAFASITGRYLTNSPEFPTSTFSINHLLQPFGKSLQKFPCHSLVIRPNPASFIVRCSTLLQRRWL
19 AB624361.1      100.0%  97.3%      PTHLDFHKTLPSLIHRAFASITGRYITNSPEFPTSTFSINHFLQPFGKSLQKFPCHSLVIRPNPASFIVRCSTLLERRWL
   consensus/100%                     PTHLDFHKTLPSLIHRAFASITGpYlTNSPEFPTShFSINHhLQPFGKS.QKFPpHSLVIpPNPsSFIVRCSTLLpR+WL
   consensus/90%                      PTHLDFHKTLPSLIHRAFASITGRYlTNSPEFPTSTFSINHLLQPFGKSLQKFPpHSLVIpPNPsSFIVRCSTLLpR+WL
   consensus/80%                      PTHLDFHKTLPSLIHRAFASITGRYlTNSPEFPTSTFSINHLLQPFGKSLQKFPpHSLVIRPNPASFIVRCSTLLpR+WL
   consensus/70%                      PTHLDFHKTLPSLIHRAFASITGRYLTNSPEFPTSTFSINHLLQPFGKSLQKFPpHSLVIRPNPASFIVRCSTLLQR+WL

                   cov     pid   481            .         5         .         .         .         .         :         . 560
 1 DRR068690       100.0% 100.0%      LLGLSALTGVWAYYHLRRGNSPQEKSDAYLSYFHPDPWRLTIRTSNVMAIPHSFFPDSGYSAPSPSSPPSPDPIVIEQPS
 2 DRR068691       100.0%  96.8%      LLGLSALTGVWAYYHLRRGNSPQEKSDAYLSYFHPDPWRLTIRTSTVTAIPHSFFPDSGYSTPSPSRPPSPHPIVIEQPS
 3 DRR006265       100.0%  97.2%      LLGLSALTGVWAYYHLRRSNSPQEKSDAYLSYFHPDPWRLTIRTSNVTAIPHSFFPDSGYSTPSPSRPPSPDPIVIKQPS
 4 DRR039989        99.9%  97.1%      LLGLSALTGVWAYYHLRRSNSPQEKSDAYLSYFHPDPWRLTIRTSNVTAIPHSFFPDSGYSTPSPSTPPSPDPIVIKQPS
 5 DRR079253        99.9%  97.2%      LLGLSALTGVWAYYHLRRSNSPQEKSDAYLSYFHPDPWRLTIRTSNVTAIPHSFFPDSGYSTPSPSRPPSPDPIVIKQPS
 6 SRR6324419-24   100.0%  95.4%      LLGLSALTGVWAYYHLRRGNSPQEKSDAYLSYFHPDPWSLTIRTSNVTAVPHLFFPDSGYSTPSPSRPPSPHPIVIEQPS
 7 SRR11781560-1   100.0%  95.5%      LLGLSALTGVWAYYHLRRGNSPQEKSDAYLSYFHPDPWSLTIRTSNVTAIPHSFFPDSGYSTPSPSRPPSPHPIVIEQPS
 8 SRR5458683      100.0%  97.2%      LLGLSALTGVWAYYHLRRSNSPQEKSDAYLSYFHPDPWRLTIRTSNVTAIPHSFFPDSGYSTPSPSRPPSPDPIVIKQPS
 9 DRR023337+40    100.0%  97.1%      LLGLSALTGVWAYYHLRRSNSPQEKSDAYLSYFHPDPWRLTIRTSNVTAIPHSFFPDSGYSTPSPSRPPSPDPIVIKQPS
10 SRR1333837-38   100.0%  97.2%      LLGLSALTGVWAYYHLRRSNSPQEKSDAYLSYFHPDPWRLTIRTSSVTAIPHSFFPDSGYSTPSPSRPPSPDPIVIKQPS
11 DRR241076       100.0%  98.5%      LLGLSALTGVWAYYHLRRSNSPQEKSDAYLSYFHPDPWRLTIRTSNVTAIPHSFFPDSGYSAPSPSSPPSPHPIVIEQPS
12 SRR17258733     100.0%  97.3%      LLGLSALTGVWAYYHLRRSNSPQEKSDAYLSYFHPDPWRLTIRTSNVTAIPHSFFPDSGYSTPSPSRPPSPDPIVIKQPS
13 SRR24542361     100.0%  96.1%      LLGLSALTGIWAYYHLRRGNSPQEKSDAYLSYFHPDPWRLTIRTSNVMAIPHSFFPDSGYSTPSPSTQPSPEPIVIEQPS
14 SRR17258732     100.0%  95.7%      LLGLSALTGIWAYYHLRRGNSPQEKSDAYLSYFHPDPWRLTIRTSNVMAIPHSFFPDSGNSTPSPSTQPSPDPIVIEQPS
15 SRR9685281      100.0%  97.2%      LLGLSALTGVWAYYHLRRSNSPQEKSDAYLSYFHPDPWRLTIRTSNVTAIPHSFFPDSGYSTPSPSRPPSPDPIVIKQPS
16 DRR035925       100.0%  99.9%      LLGLSALTGVWAYYHLRRGNSPQEKSDAYLSYFHPDPWRLTIRTSNVMAIPHSFFPDSGYSAPSPSSPPSPDPIVIEQPS
17 AB186123.1       99.9%  97.5%      LLGLSALTGVWAYYHLRRGNSPQEKSDAYLSYFHPDPWRLTIRTSNVMAIPHSFFPDSGYSTSSPSRPPSPHPIVIEQPS
18 KJ433990        100.0%  97.5%      LLGLSALTGVWAYYHLRRGNSPQEKSDAYLSYFHPDPWRLTIRTSNVMAIPHSFFPDSGYSTPSPSRPPSSDPIVIEQPS
19 AB624361.1      100.0%  97.3%      LLGLSALTGVWAYYHLRRGNSPQEKSDAYLSYFHPDPWRLTIRTSNVMAIPHSFFPDSGYSAPSPSSPPSPDPIVIAQPS
   consensus/100%                     LLGLSALTGlWAYYHLRRuNSPQEKSDAYLSYFHPDPWpLTIRTSsVhAlPH.FFPDSG.SssSPSp.PSscPIVItQPS
   consensus/90%                      LLGLSALTGlWAYYHLRRuNSPQEKSDAYLSYFHPDPWpLTIRTSsVhAIPHSFFPDSGYSsPSPSp.PSPcPIVIcQPS
```

```
   consensus/80%                    LLGLSALTGVWAYYHLRRuNSPQEKSDAYLSYFHPDPWRLTIRTSNVhAIPHSFFPDSGYSsPSPSpPPSPcPIVIcQPS
   consensus/70%                    LLGLSALTGVWAYYHLRRuNSPQEKSDAYLSYFHPDPWRLTIRTSNVhAIPHSFFPDSGYSTPSPSpPPSP-PIVIcQPS

                      cov     pid  561            .              .              .             6             .              .              .             . 640
 1 DRR068690        100.0% 100.0%      SNVSKHHQVQNQAYLTPAHPETPSSPGKQEIESGETLATGFPNVATLSGPSKASKPDAPPTPLSSDPTAHGPILPHKELF
 2 DRR068691        100.0%  96.8%      ANVSKHHQVQNQAHLTPAHSETPPSPTKQEIKSGETLATGFPNVATLSGPSEASKPDTPPTPLSSDPTAHGPILPHKELF
 3 DRR006265        100.0%  97.2%      SNVSKHHQVQNQAYLTPAHPETPPSPSKQEIKSGETLATGFPNVATLSGPSEASKPDAPPTPLNSDPTAHGPILPHKELF
 4 DRR039989         99.9%  97.1%      SNVSKHHQVQNQAYLTPAHPETPPSPSKQEIKSGETLATGFPNVATLSGPSEASKPDAPPTPLNSDPTAHGPILPHKELF
 5 DRR079253         99.9%  97.2%      SNVSKHHQVQNQAYLTPAHPETPPSPSKQEIKSGETLATGFPNVATLSGPSEASKPDAPPTPLNSDPTAHGPILPHKELF
 6 SRR6324419-24    100.0%  95.4%      SDVSKHHQVQNQAYLTPAHPETPPSPTKQEIKSGETLATGFPNVATLNGPSEASKPDAPPTPLSSDSTAHGPILPHKELF
 7 SRR11781560-1    100.0%  95.5%      SDVSKHHQVQNQAYLTPAHPETPPSPTKQEIKSGETLATGFPNVATLNGPSEASKPDAPPTPLSSDSTAHGPILPHKELF
 8 SRR5458683       100.0%  97.2%      SNVSKHHQVQNQAYLTPAHPETPPSPSKQEIKSGETLATGFPNVATLSGPSEASKPDAPPTPLNSDPTAHGPILPHKELF
 9 DRR023337+40     100.0%  97.1%      SNVSKHHQVQNQAYLTPAHPETPPSPSKQEIKSGETLATGFPNVATLSGPSEASKPDAPPTPLNSDPTAHGPILPHKELF
10 SRR1333837-38    100.0%  97.2%      SNVSKHHQVQNQAYLTPAHPETPPSPSKQEIKSGETLATGFPNVATLSGPSEASKPDAPPTPLNSDPTAHGPILPHKELF
11 DRR241076        100.0%  98.5%      SNVSKHHQVQNQAYLTPAHPETPPCPSKQKIESGETLATGFPNVATLSGPSEASKPDAPPTPLSSDPTAHGPILPHRELF
12 SRR17258733      100.0%  97.3%      SNVSKHHQVQNQAYLTPAHPETPPSPSKQEIKSGETLATGFPNVATLSGPSEASKPDAPPTPLNSDPTAHGPILPHKELF
13 SRR24542361      100.0%  96.1%      SNVSKRHLVQNQAYLTPAHPETPPSPSKQEIKSGETLATGFPNVATLSGPSEASKPDAPPTPLSSDPTAHGPILPHKELF
14 SRR17258732      100.0%  95.7%      SNVSKRHLVPNQAYPTPAHPETPPSPSKQEIKSGETLATGFPNVATLSGPSEASKPDAPPTPLSSDPTAHGPILPHKELF
15 SRR9685281       100.0%  97.2%      SNVSKHHQVQNQAYLTPAHPETPPSPSKQEIKSGETLATGFPNVATLSGPSEASKPDAPPTPLNSDPTAHGPILPHKELF
16 DRR035925        100.0%  99.9%      SNVSKHHQVQNQAYLTPAHPETPSSPGKQEIESGETLATGFPNVATLSGPSKASKPDAPPTPLSSDPTAHGPILPHKELF
17 AB186123.1        99.9%  97.5%      SNVSKHHQVQNQAYLTPAHPETPPSPSKQEIKSGETLATGFPNVATLSGPSEASKPDAPPTPLSSDPTAHGPILPHKELF
18 KJ433990         100.0%  97.5%      SNVSKHHQVQNQAHLTPAHPETPPSPSKQEIKSGETLATGFPNVATLSGPSEASKPDAPPTPLSSDPTAHGPILPHKELF
19 AB624361.1       100.0%  97.3%      SNVSKHHQVQNQAYLTPAHPETPPSPSKQEIKSGETLATGFPNVATLSGPSEASKPDAPPTPLSSDPTAHGPILPHKELF
   consensus/100%                      usVSK+H.V.NQAa.TPAHsETPssPsKQcIcSGETLATGFPNVATLsGPScASKPDsPPTPLsSDsTAHGPILPH+ELF
   consensus/90%                       SsVSK+H.VQNQAaLTPAHPETPsSPsKQEIcSGETLATGFPNVATLsGPScASKPDAPPTPLsSDsTAHGPILPHKELF
   consensus/80%                       SNVSKHHQVQNQAYLTPAHPETPPSPoKQEIKSGETLATGFPNVATLSGPSEASKPDAPPTPLsSDPTAHGPILPHKELF
   consensus/70%                       SNVSKHHQVQNQAYLTPAHPETPPSPSKQEIKSGETLATGFPNVATLSGPSEASKPDAPPTPLsSDPTAHGPILPHKELF

                      cov     pid  641            :              .              .             .             .              7              .             . 720
 1 DRR068690        100.0% 100.0%      GISSPDHECTFLNRKRLNVSTLPFPAQPCLLVAFAQASNSTPQRVWAHLCTLFPDSLLDGPLERSQGFSSEHLEALAWSL
 2 DRR068691        100.0%  96.8%      GISSPDHECTFLNRKRLHVSTLPFPAQPCLLVAFAQASNSTPQRVWAHLCTLFPDSLLDGPLERSQGFSSEHLEALAWSL
 3 DRR006265        100.0%  97.2%      GISSPDHECTFLNRKRLNVSTLPFPAQPCLLVAFAQASNSTPQRVWDHLCTLFPDSLLDGPLERSQGFSSEHLEALAWSL
 4 DRR039989         99.9%  97.1%      GISSPDHECTFLNRKRLNVSTLPFPAQPCLLVAFAQASNSTPQRVWDHLCTLFPDSLLDGPLERSQGFSSEHLEALAWSL
 5 DRR079253         99.9%  97.2%      GISSPDHECTFLNRKRLNVSTLPFPAQPCLLVAFAQASNSTPQRVWDHLCTLFPDSLLDGPLERSQGFSSEHLEALAWSL
 6 SRR6324419-24    100.0%  95.4%      GISSPDHECTFLQGKRLNVSKLPFPAQPCLLVAFAQASNSTPQRVWAHLCTLFPDSLLDGPLERSQGFSSEHLEALAWSL
 7 SRR11781560-1    100.0%  95.5%      GISSPDHECTFLQVKRLNVSKLPFPAQPCLLVAFAQASNSTPQRVWAHLCTLFPDSLLDGPLERSQGFSSEHLEALAWSL
 8 SRR5458683       100.0%  97.2%      GISSPDHECTFLNRKRLNVSTLPFPAQPCLLVAFAQASNSTPQRVWDHLCTLFPDSLLDGPLERSQGFSSEHLEALAWSL
 9 DRR023337+40     100.0%  97.1%      GISSPDHECTFLNRKRLNVSTLPFPAQPCLLVAFAQASNSTPQRVWDHLCTLFPDSLLDGPLERSQGFSSEHLEALAWSL
10 SRR1333837-38    100.0%  97.2%      GISSPDHECTFLNRKRLNVSTLPFPAQPCLLVAFAQASNSTPQRVWDHLCTLFPDSLLDGPLERSQGFSSEHLEALAWSL
11 DRR241076        100.0%  98.5%      GISSPDHECTFLNRKRLNVSTLPFPAQPCLLVAFAQASNSTPQRVWAHLCTLFPDSLLDGPLERSQGFSSEHLEALAWSL
12 SRR17258733      100.0%  97.3%      GISSPDHECTFLNRKRLNVSTLPFPAQPCLLVAFAQASNSTPQRVWDHLCTLFPDSLLDGPLERSQGFSSEHLEALAWSL
13 SRR24542361      100.0%  96.1%      GISSPDHECTFLNRKRLNVSTLPFPDQPCLLVAFAQASNSTPQQVWAHLCTLFPDSLLDGPLERSQGFSSEHLEALAWSL
14 SRR17258732      100.0%  95.7%      GISSPDHECTFLNRKRLNVSTLPFPDQPCLLVAFAQASNSTPQQVWAHLCTLFPDSLLDGPLERSQGFSSEHLEALAWSL
15 SRR9685281       100.0%  97.2%      GISSPDHECTFLNRKRLNVSTLPFPAQPCLLVAFAQASNSTPQRVWDHLCTLFPDSLLDGPLERSQGFSSEHLEALAWSL
```

```
16 DRR035925        100.0%  99.9%       GISSPDHECTFLNRKRLNVSTLPFPAQPCLLVAFAQASNSTPQRVWAHLCTLFPDSLLDGPLERSQGFSSEHLEALAWSL
17 AB186123.1        99.9%  97.5%       GISSPDHECTFLNRKRLNVSMLPFPAQPCLLVAFAQASNSTPQRVWAHLCTLFPDSLLDGPLERSQGFSSEHLEALAWSL
18 KJ433990         100.0%  97.5%       GISSPDHECTFLNRKRLNVSTLPFPAQPCLLVAFAQASNSTPQRVWDHLCTLFPDSLLDGPLERSQGFSSEHLEALAWSL
19 AB624361.1       100.0%  97.3%       GISSPDHECTFLNRKRLNVSTLPFPAQPCLLVAFAQASNSTPQRVWAHLCTLFPDSLLDGPLERSQGFSSEHLEALAWSL
   consensus/100%                       GISSPDHECTFLphKRLpVShLPFPsQPCLLVAFAQASNSTPQpVWsHLCTLFPDSLLDGPLERSQGFSSEHLEALAWSL
   consensus/90%                        GISSPDHECTFLptKRLNVSpLPFPsQPCLLVAFAQASNSTPQpVWsHLCTLFPDSLLDGPLERSQGFSSEHLEALAWSL
   consensus/80%                        GISSPDHECTFLNRKRLNVSTLPFPAQPCLLVAFAQASNSTPQRVWsHLCTLFPDSLLDGPLERSQGFSSEHLEALAWSL
   consensus/70%                        GISSPDHECTFLNRKRLNVSTLPFPAQPCLLVAFAQASNSTPQRVWsHLCTLFPDSLLDGPLERSQGFSSEHLEALAWSL

                      cov    pid   721           .         .         :         .         .         .         .        8 800
 1 DRR068690        100.0% 100.0%       NYRVSYRHGEHLNTIGPDDAPLLSLIYTGDGSIGHWAADDSPLSAPLSPIRGSAKTLNSFASTAIRFRDSHGNLLPFRQV
 2 DRR068691        100.0%  96.8%       NYRVSYRHGEHVNTIGPDDAPLLRLIYTGDRSIGHWAADESPLSPPPSLIRGSAKTLNSFSSTAIRFRDSYGNLLPFRQV
 3 DRR006265        100.0%  97.2%       NYRVSYRHGEHVNTIGPDDAPLLRLIYTGDGSIGHWAADESPLSPPPSHIRGSARTLNSFASTAIRFRDSHGNLLPFRQV
 4 DRR039989         99.9%  97.1%       NYRVSYRHGEHVNTIGPDDAPLLRLIYTGDGSIGHWAADESPLSPPPSHIRGSARTLNSFASTAIRFRDSHGNLLPFRQV
 5 DRR079253         99.9%  97.2%       NYRVSYRHGEHVNTIGPDDAPLLRLIYTGDGSIGHWAADESPLSPPPSHIRGSARTLNSFASTAIRFRDSHGNLLPFRQV
 6 SRR6324419-24    100.0%  95.4%       NYRVSYRHDEHVNTIGPDDAPLLRLIYTGDGSIGHWAADESPLSPPPSLIRGSAKTLNSFSSAAIRFRDSHGNFLPFRQV
 7 SRR11781560-1    100.0%  95.5%       NYRVSYRHDEHVNTIGPDDAPLLRLIYTGDGSIGHWAADESPLSPPPSLIRGSAKTLNSFSSAAIRFRDSHGNFLPFRQV
 8 SRR5458683       100.0%  97.2%       NYRVSYRHGEHVNTIGPDDAPLLRLIYTGDGSIGHWAADESPLSPPPSHIRGSARTLNSFASTAIRFRDSHGNLLPFRQV
 9 DRR023337+40     100.0%  97.1%       NYRVSYRHGEHVNTIGPDDAPLLRLIYTGDGSIGHWAADESPLSPPPSHIRGSARTLNSFASTAIRFRDSHGNLLPFRQV
10 SRR1333837-38    100.0%  97.2%       NYRVSYRHGEHVNTIGPDDAPLLRLIYTGDGSIGHWAADESPLSPPPSHIRGSARTLNSFASTAIRFRDSHGNLLPFRQV
11 DRR241076        100.0%  98.5%       NYRVSYRHGERLNTIGPDDAPLLSLIYTGDGSIGHWAADDSPPSAPLSPIRGSAKTLNSFASTAIRFRDSHGNLLPFRQV
12 SRR17258733      100.0%  97.3%       NYRVSYRHGEHVNTIGPDDAPLLRLIYTGDGSIGHWAADESPLSPPPSHIRGSARTLNSFASTAIRFRDSHGNLLPFRQV
13 SRR24542361      100.0%  96.1%       NYHVSYRHGEHVNTIGPDDAPLLRLIYTGDGSIGHWAADESPLSPPLSSIRGSAKTLNSFASTAIRFRDSHGNLLPFRQV
14 SRR17258732      100.0%  95.7%       NYHVSYRHGEHVNTIGPDDAPLLRLIYTGDGSIGHWAADESPLSPPLSSIRGSAKTLNSFASTAIRFRDSHGNLLPFRQV
15 SRR9685281       100.0%  97.2%       NYRVSYRHGEHVNTIGPDDAPLLRLIYTGDGSIGHWAADESPLSPPPSHIRGSARTLNSFASTAIRFRDSHGNLLPFRQV
16 DRR035925        100.0%  99.9%       NYRVSYRHGEHLNTIGPDDAPLLSLIYTGDGSIGHWAADDSPLSAPLSPIRGSAKTLNSFASTAIRFRDSHGNLLPFRQV
17 AB186123.1        99.9%  97.5%       NYRVSYRHGEHLNTIGPDDAPLLSLIYTGDGSIGHWAADESPLSPPPSPIRGSAKTLNSFASTAIRFRDSHGNLLPFRQV
18 KJ433990         100.0%  97.5%       NYRVSYRHGEHLNTIGPDDAPLLSLIYTGDGSIGHWAADESPLSPPLSPIRGSAKALNSFASTAIRFRDSHGNLLPFRQV
19 AB624361.1       100.0%  97.3%       NYRVSYRHGEHLNTIGPDDAPLLSLIYTGDGSIGHWATDESPLSAPLSPIRGSAKTLNSFASTAVRFRDSHGNLLPFRQV
   consensus/100%                       NY+VSYRHsE+lNTIGPDDAPLLpLIYTGDtSIGHWAsD-SP.SsP.S.IRGSA+sLNSFuSsAlRFRDSaGNhLPFRQV
   consensus/90%                        NY+VSYRHsEHlNTIGPDDAPLLpLIYTGDGSIGHWAAD-SPLSsP.S.IRGSA+TLNSFuSsAIRFRDSHGNhLPFRQV
   consensus/80%                        NYRVSYRHGEHlNTIGPDDAPLLpLIYTGDGSIGHWAADESPLSsP.S.IRGSA+TLNSFASTAIRFRDSHGNLLPFRQV
   consensus/70%                        NYRVSYRHGEHlNTIGPDDAPLLpLIYTGDGSIGHWAADESPLSPP.S.IRGSA+TLNSFASTAIRFRDSHGNLLPFRQV

                      cov    pid   801           .         .         .         .         :         .         .         . 880
 1 DRR068690        100.0% 100.0%       HEYTLCKPRAKNLASNMKNETDGVIQSTLRTASSDPTFFHRLDQRADFPPQVSVQLIHIAGFPGCGKTFPVTQLLKTKAF
 2 DRR068691        100.0%  96.8%       HEYKLCKPRAKNLASNMKNETDGVIQSNLRAASSDPTFFHRLDQRADFAPQVSVQLIHITGFPGCGKTFPVTQLLKTKAF
 3 DRR006265        100.0%  97.2%       HEYKLCKPRAKNLASNMKNETDGVIQSSLRTASSDPTFFHRLDQRADFAPQVSVQLIHITGFPGCGKTFPVTQLLKTKAF
 4 DRR039989         99.9%  97.1%       HEYKLCKPRAKNLASNM-NETDGVIQSSLRTASSDPTFFHRLDQRADFAPQVSVQLIHITGFPGCGKTFPVTQLLKTKAF
 5 DRR079253         99.9%  97.2%       HEYKLCKPRAKNLASNMKNETDGVIQSSLRTASSDPTFFHRLDQRADFAPQVSVQLIHITGFPGCGKTFPVTQLLKTKAF
 6 SRR6324419-24    100.0%  95.4%       HEYKLCKPRAKNLASNMKNETDGVIQSNLRAASSDPTFFHRLDQRADLFLQVSVQLIHITGFPGCGKTFPVTQLLKTKGL
 7 SRR11781560-1    100.0%  95.5%       HEYKLCKPRAKNLASNMKNETDGVIQSNLRAASSDPTFFHRLDQRADTFLQVSVQLIHITGFPGCGKTFPVTQLLKTKGL
 8 SRR5458683       100.0%  97.2%       HEYKLCKPRAKNLASNMKNETDGVIQSSLRTASSDPTFFHRLDQRADFAPQVSVQLIHITGFPGCGKTFPVTQLLKTKAF
 9 DRR023337+40     100.0%  97.1%       HEYKLCKPRAKNLASNMKNETDGVIQSSLRTASSDPTFFHRLDQRADFAPQVSVQLIHITGFPGCGKTFPVTQLLKTKAF
```

```
10 SRR1333837-38   100.0%  97.2%       HEYKLCKPRAKNLASNMKNETDGVIQSSLRTASSDPTFFHRLDQRADFAPQVSVQLIHITGFPGCGKTFPVTQLLKTKAF
11 DRR241076       100.0%  98.5%       HEYTLCKPRAKNLASNMKNETDGVIQSTLRTASSDPTFFHRLDQRADFAPQVSVQLIHITGFPGCGKTFPVTQLLKTKAF
12 SRR17258733     100.0%  97.3%       HEYKLCKPRAKNLASNMKNETDGVIQSSLRTASSDPTFFHRLDQRADFAPQVSVQLIHITGFPGCGKTFPVTQLLKTKAF
13 SRR24542361     100.0%  96.1%       HEYKLCKPRAKNLASNMKNETDGVIQSSLRAASSDPTFFQRLDQRADFAPQVSVQLIHITGFPGCGKTFPVTQLLKTKAF
14 SRR17258732     100.0%  95.7%       HEYKLCKPRAKNLASNMKNETDGVIQSSLRAASSDPTFFQRLDQRADFAPQVSVQLIHITGFPGCGKTFPVTQLLKTKAF
15 SRR9685281      100.0%  97.2%       HEYKLCKPRAKNLASNMKNETDGVIQSSLRTASSDPTFFHRLDQRADFAPQVSVQLIHITGFPGCGKTFPVTQLLKTKAF
16 DRR035925       100.0%  99.9%       HEYTLCKPRAKNLASNMKNETDGVIQSTLRTASSDPTFFHRLDQRADFPPQVSVQLIHIAGFPGCGKTFPVTQLLKTKAF
17 AB186123.1       99.9%  97.5%       HEYKLCKPRAK-IWPQHENETDGVIQSSLRAASSDPTFFHRLDQRADFAPQVSVQLIHITGFPGCGKTFPVTQLLKTKAF
18 KJ433990        100.0%  97.5%       HEYKLCKPRAKNLASNMKNETDGVIQSSLRAASSDPTFFHRLDQRADFAPRVSVQLIHITGFPGCGKTFPVTQLLKTKAF
19 AB624361.1      100.0%  97.3%       HEYTLCKPRAKNLASNMKNETDGVIQSTLRAASSDPTFFHRLDQRADFAPQVSVQLIHITGFPGCGKTFPVTQLLKTKAF
   consensus/100%                      HEYpLCKPRAK.lhsph.NETDGVIQSsLRsASSDPTFFpRLDQRADh..pVSVQLIHIsGFPGCGKTFPVTQLLKTKuh
   consensus/90%                       HEYpLCKPRAKNLASNMcNETDGVIQSsLRsASSDPTFFpRLDQRADh..QVSVQLIHIsGFPGCGKTFPVTQLLKTKuh
   consensus/80%                       HEYpLCKPRAKNLASNMKNETDGVIQSoLRsASSDPTFFHRLDQRADFsPQVSVQLIHITGFPGCGKTFPVTQLLKTKAF
   consensus/70%                       HEYKLCKPRAKNLASNMKNETDGVIQSoLRsASSDPTFFHRLDQRADFAPQVSVQLIHITGFPGCGKTFPVTQLLKTKAF

                      cov     pid  881          .         9         .         .         .         .         :         . 960
 1 DRR068690       100.0% 100.0%       KGQYRVAVPTTELRSEWKDHMKLPSSEVWRVSTWETSLMKSAPVLVIDEVYKMPRGFLDLALVADPALQFVILLGDPCQT
 2 DRR068691       100.0%  96.8%       KGQYRVAVPTTELRSEWKDHMKLPSSDVWRVSTWETSLMKSAPVLVIDEVYKMPRGFLDLALVADPALQFVILLGDPCQT
 3 DRR006265       100.0%  97.2%       KGQYRVAVPTTELRSEWKDHMKLPSSDAWRVSTWETSLMKSAPVLVIDEVYKMPRGFLDLALVADPALQFVILLGDPCQT
 4 DRR039989        99.9%  97.1%       KGQYRVAVPTTELRSEWKDHMKLPSSDAWRVSTWETSLMKSAPVLVIDEVYKMPRGFLDLALVADPALQFVILLGDPCQT
 5 DRR079253        99.9%  97.2%       KGQYRVAVPTTELRSEWKDHMKLPSSDAWRVSTWETSLMKSAPVLVIDEVYKMPRGFLDLALVADPALQFVILLGDPCQT
 6 SRR6324419-24   100.0%  95.4%       KEQYRVTVPTTELRSEWKDHMKLPSSDAWRVSTWETSLMKSAPVLVIDEVYKMPRGFLDLALVADPALQFVILLGDPCQT
 7 SRR11781560-1   100.0%  95.5%       KEQYRVTVPTTELRSEWKDHMKLPSSDAWRVSTWETSLMKSAPVLVIDEVYKMPRGFLDLALVADPALQFVILLGDPCQT
 8 SRR5458683      100.0%  97.2%       KGQYRVAVPTTELRSEWKDHMKLPSSDAWRVSTWETSLMKSAPVLVIDEVYKMPRGFLDLALVADPALQFVILLGDPCQT
 9 DRR023337+40    100.0%  97.1%       KGQYRVAVPTTELRSEWKDHMKLPSSDAWRVSTWETSLMKSAPVLVIDEVYKMPRGFLDLALVADPALQFVILLGDPCQT
10 SRR1333837-38   100.0%  97.2%       KGQYRVAVPTTELRSEWKDHMKLPSSDAWRVSTWETSLMKSAPVLVIDEVYKMPRGFLDLALVADPALQFVILLGDPCQT
11 DRR241076       100.0%  98.5%       KGQYRVAVPTTELRSEWKDHMKLPSSEVWRVSTWETSLMKSAPVLIIDEVYKMPRGFLDLPLLADPALQFVILLGDPCQT
12 SRR17258733     100.0%  97.3%       KGQYRVAVPTTELRSEWKDHMKLPSSDAWRVSTWETSLMKSAPVLVIDEVYKMPRGFLDLALVADPALQFVILLGDPCQT
13 SRR24542361     100.0%  96.1%       KSQYRVAVPTTELRSEWKDHMKLPASDAWRVSTWETSLMKSAPVLVIDEVYKMPRGFLDLALLADPAIQFVILLGDPCQT
14 SRR17258732     100.0%  95.7%       KSQYRVAVPTTELRTEWKDHMKLPASDVWRVSTWETSLMKSAPVLVIDEVYKMPRGFLDLALLADPAIQFVILLGDPCQT
15 SRR9685281      100.0%  97.2%       KGQYRVAVPTTELRSEWKDHMKLPSSDAWRVSTWETSLMKSAPVLVIDEVYKMPRGFLDLALVADPALQFVILLGDPCQT
16 DRR035925       100.0%  99.9%       KGQYRVAVPTTELRSEWKDHMKLPSSEVWRVSTWETSLMKSAPVLVIDEVYKMPRGFLDLALVADPALQFVILLGDPCQT
17 AB186123.1       99.9%  97.5%       KGQYRVAVPTTELRSEWKDHMKLPSSDAWRVSTWETSLMKSAPVLVIDEVYKMPRGFLDLALVADPALQFVILLGDPCQT
18 KJ433990        100.0%  97.5%       KGQYRVAVPTTELRSEWKDHMKLPSSDVWRVSTWETSLMKSAPVLVIDEVYKMPRGFLDLALVADPALQFVILLGDPCQT
19 AB624361.1      100.0%  97.3%       KGQYRVAVPTTELRSEWKDHMKLPSSEVWRVSTWETSLMKSAPVLVIDEVYKMPRGFLDLALVADPALQFVILLGDPCQT
   consensus/100%                      KtQYRVsVPTTELRoEWKDHMKLPuS-sWRVSTWETSLMKSAPVLlIDEVYKMPRGFLDLsLlADPAlQFVILLGDPCQT
   consensus/90%                       KtQYRVsVPTTELRSEWKDHMKLPuS-sWRVSTWETSLMKSAPVLVIDEVYKMPRGFLDLALlADPAlQFVILLGDPCQT
   consensus/80%                       KuQYRVAVPTTELRSEWKDHMKLPSS-sWRVSTWETSLMKSAPVLVIDEVYKMPRGFLDLALVADPALQFVILLGDPCQT
   consensus/70%                       KGQYRVAVPTTELRSEWKDHMKLPSSDsWRVSTWETSLMKSAPVLVIDEVYKMPRGFLDLALVADPALQFVILLGDPCQT

                      cov     pid  961          .         .         .         0         .         .         .         . 1040
 1 DRR068690       100.0% 100.0%       VYSSVNPDSSNYRLLSEVEHLKPYRDFYCHWTHRLPRRLAQFFGVSTTNPQEGFIGRRDNPHKSYPILTSSQQTARVCAG
 2 DRR068691       100.0%  96.8%       VYSSVNPDSSNYRLISEVEHLKPYRDFYCHWTHRLPRRLARFFGVSTTNPQEGFIGRRDNPHKSYPILTSSQQTARVCAG
 3 DRR006265       100.0%  97.2%       VYSSVNPDSSNYRLISEVEHLKPYRDFYCHWTHRLPLRLARFFGVSTTNPQEGFIGRRDNPHKSYPILTSSQQTARVCAG
```

```
 4 DRR039989         99.9%  97.1%     VYSSVNPDSSNYRLISEVEHLKPYRDFYCHWTHRLPLRLARFFGVSTTNPQEGFIGRRDNPHKSYPILTSSQQTARVCAG
 5 DRR079253         99.9%  97.2%     VYSSVNPDSSNYRLISEVEHLKPYRDFYCHWTHRLPLRLARFFGVSTTNPQEGFIGRRDNPHKSYPILTSSQQTARVCAG
 6 SRR6324419-24    100.0%  95.4%     VYSSVNPDSSNYRLISEVEHLKPYRDFYCHWTHRLPRRLARFFGVSTTNPQEGFIGRRDNPHKSYPILTSSQQTARVCAG
 7 SRR11781560-1    100.0%  95.5%     VYSSVNPDSSNYRLISEVEHLKPYRDFYCHWTHRLPRRLARFFGVSTTNPQEGFIGRRDNPHKSYPILTSSQQTARVCAG
 8 SRR5458683       100.0%  97.2%     VYSSVNPDSSNYRLISEVEHLKPYRDFYCHWTHRLPLRLARFFGVSTTNPQEGFIGRRDNPHKSYPILTSSQQTARVCAG
 9 DRR023337+40     100.0%  97.1%     VYSSVNPDSSNYRLISEVEHLKPYRDFYCHWTHRLPLRLARFFGVSTTNPQEGFIGRRDNPHKSYPILTSSQQTARVCAG
10 SRR1333837-38    100.0%  97.2%     VYSSVNPDSSNYRLISEVEHLKPYRDFYCHWTHRLPLRLARFFGVSTTNPQEGFIGRRDNPHKSYPILTSSQQTARVCAG
11 DRR241076        100.0%  98.5%     VYSSVNPDSSNYRLLSEVEHLKPYRDFYCHWTHRLPRRLARFFGVSTTNPQEGFIGRRDNPHKSYPILTSSQQTARVCAG
12 SRR17258733      100.0%  97.3%     VYSSVNPDSSNYRLISEVEHLKPYRDFYCHWTHRLPLRLARFFGVSTTNPQEGFIGRRDNPHKSYPILTSSQQTARVCAG
13 SRR24542361      100.0%  96.1%     VYSSVNPDSSNYRLISEVEHLKPYRDFYCLWTHRLPLRLARFFGVSTTNPQEGFIGRRDNPHKSYPILTSSQQTARVCAG
14 SRR17258732      100.0%  95.7%     VYSSVNPDSSNYRLISEVEHLKPYRDFYCLWTHRLPLRLARFFGVSTTNPQEGFIGRRDNPHKSYPILTSSQQTARVCAG
15 SRR9685281       100.0%  97.2%     VYSSVNPDSSNYRLISEVEHLKPYRDFYCHWTHRLPLRLARFFGVSTTNPQEGFIGRRDNPHKSYPILTSSQQTARVCAG
16 DRR035925        100.0%  99.9%     VYSSVNPDSSNYRLLSEVEHLKPYRDFYCHWTHRLPRRLAQFFGVSTTNPQEGFIGRRDNPHKSYPILTSSQQTARVCAG
17 AB186123.1        99.9%  97.5%     VYSSVNPDSSNYRLISEVEHLKPYRDFYCHWTHRLPLRLARFFGVSTTNPQEGFIGRRDNPHKSYPILTSSQQTARVCAG
18 KJ433990         100.0%  97.5%     VYSSVNPDSSNYRLISEVEHLKPYRDFYCHWTHRLPLRLARFFGVSTTNPQEGFIGRRDNPHKSYPTLTSSQQTARVCAG
19 AB624361.1       100.0%  97.3%     VYSSVNPDSSNYRLLSEVEHLKPYRDFYCHWTHRLPRRLARFFGVSTTNPQEGFIGRRDNPHKSYPILTSSQQTARVCAG
   consensus/100%                     VYSSVNPDSSNYRLlSEVEHLKPYRDFYChWTHRLPhRLApFFGVSTTNPQEGFIGRRDNPHKSYPhLTSSQQTARVCAG
   consensus/90%                      VYSSVNPDSSNYRLlSEVEHLKPYRDFYChWTHRLPhRLApFFGVSTTNPQEGFIGRRDNPHKSYPILTSSQQTARVCAG
   consensus/80%                      VYSSVNPDSSNYRLlSEVEHLKPYRDFYCHWTHRLPhRLARFFGVSTTNPQEGFIGRRDNPHKSYPILTSSQQTARVCAG
   consensus/70%                      VYSSVNPDSSNYRLISEVEHLKPYRDFYCHWTHRLPhRLARFFGVSTTNPQEGFIGRRDNPHKSYPILTSSQQTARVCAG

                       cov    pid 1041         :         .         .         .         .         1         .         . 1120
 1 DRR068690        100.0% 100.0%     TGHRALTFCSSQGSTFQAPAQIFVDSNVASVHVSATLVATTRSRSGVIFTGNHRLFQSRPGTAPLFEAMLNDQPFNFLNT
 2 DRR068691        100.0%  96.8%     TGHRALTFCSSQGSTFQAPAQIFVDSNVASVHVSATLVATTRSRSGVIFTGNHRLFQSRPGTAPLFEAMLNDQPFNFLNT
 3 DRR006265        100.0%  97.2%     TGHRALTFCSSQGSTFQAPAQIFVDSNVASVHVSATLVATTRSRSGVIFTGNHRLFQSRPGTAPLFEAMLNDQPFSFLNT
 4 DRR039989         99.9%  97.1%     TGHRALTFCSSQGSTFQAPAQIFVDSNVASVHVSATLVATTRSRSGVIFTGNHRLFQSRPGTAPLFEAMLNDQPFSFLNT
 5 DRR079253         99.9%  97.2%     TGHRALTFCSSQGSTFQAPAQIFVDSNVASVHVSATLVATTRSRSGVIFTGNHRLFQSRPGTAPLFEAMLNDQPFSFLNT
 6 SRR6324419-24    100.0%  95.4%     TGHRALTFCSSQGSTFHAPAQIFVDSNVASVHVSATLVATTRSRSGVIFTGNHRLFHSRPGTAPLFEAMLNDQPFNFLNT
 7 SRR11781560-1    100.0%  95.5%     TGHRALTFCSSQGSTFQAPAQIFVDSNVASVHVSATLVATTRSRSGVIFTGNHRLFQSRPGTAPLFEAMLNDQPFNFLNT
 8 SRR5458683       100.0%  97.2%     TGHRALTFCSSQGSTFQAPAQIFVDSNVASVHVSATLVATTRSRSGVIFTGNHRLFQSRPGTAPLFEAMLNDQPFSFLNT
 9 DRR023337+40     100.0%  97.1%     TGHRALTFCSSQGSTFQAPAQIFVDSNVASVHVSATLVATTRSRSGVIFTGNHRLFQSRPGTAPLFEAMLNDQPFSFLNT
10 SRR1333837-38    100.0%  97.2%     TGHRALTFCSSQGSTFQAPAQIFVDSNVASVHVSATLVATTRSRSGVIFTGNHRLFQSRPGTAPLFEAMLNDQPFSFLNT
11 DRR241076        100.0%  98.5%     TGHRALTFCSSQGSTFQAPAQIFVDSNVASVHVSATLVATTRSRSGVIFTGNHRLFQSRPGTAPLFEAMLNDQPFNFLNT
12 SRR17258733      100.0%  97.3%     TGHRALTFCSSQGSTFQAPAQIFVDSNVASVHVSATLVATTRSRSGVIFTGNHRLFQSRPGTAPLFEAMLNDQPFSFLNT
13 SRR24542361      100.0%  96.1%     TGHRALTFCSSQGSTFQAPAQIFIDSNVASVHVSATLVATTRSRSGVIFTGNHRLFQSRPGTAPLFEAMLNDQPFNFLNT
14 SRR17258732      100.0%  95.7%     TGHRALTFCSSQGSTFQAPAQIFIDSNVASVHVSATLVATTRSRSGVIFTGNHRLFQSRPGTAPLFEAMLNDQPFNFLNT
15 SRR9685281       100.0%  97.2%     TGHRALTFCSSQGSTFQAPAQIFVDSNVASVHVSATLVATTRSRSGVIFTGNHRLFQSRPGTAPLFEAMLNDQPFSFLNT
16 DRR035925        100.0%  99.9%     TGHRALTFCSSQGSTFQAPAQIFVDSNVASVHVSATLVATTRSRSGVIFTGNHRLFQSRPGTAPLFEAMLNDQPFNFLNT
17 AB186123.1        99.9%  97.5%     TGHRALTFCSSQGSTFQAPAQIFVDSNVASVHVSATLVATTRSRSGVIFTGNHRLFQSRPGTAPLFEAMLNDQPFNFLNT
18 KJ433990         100.0%  97.5%     TGHRALTFCSSQGSTFQAPAQIFVDSNVASVHVSATLVATTRSRSGVIFTGNHRLFQSRPGTAPLFEAMLNDQPFDFLNT
19 AB624361.1       100.0%  97.3%     TGHRALTFCSSQGSTFQAPAQIFVDSNVASVHVSATLVATTRSRSGVIFTGNHRLFQLRPGTAPLFEAMLNDQPFNFLNT
   consensus/100%                     TGHRALTFCSSQGSTFpAPAQIFlDSNVASVHVSATLVATTRSRSGVIFTGNHRLFp.RPGTAPLFEAMLNDQPFsFLNT
   consensus/90%                      TGHRALTFCSSQGSTFQAPAQIFlDSNVASVHVSATLVATTRSRSGVIFTGNHRLFQSRPGTAPLFEAMLNDQPFsFLNT
   consensus/80%                      TGHRALTFCSSQGSTFQAPAQIFVDSNVASVHVSATLVATTRSRSGVIFTGNHRLFQSRPGTAPLFEAMLNDQPFsFLNT
```

```
   consensus/70%                      TGHRALTFCSSQGSTFQAPAQIFVDSNVASVHVSATLVATTRSRSGVIFTGNHRLFQSRPGTAPLFEAMLNDQPFsFLNT

                          cov    pid 1121      .         .         :         .         .         .         .        2 1200
 1 DRR068690          100.0% 100.0%   FANELGGMELITSPIKERKTILRGGSYDFSAYRSYQKRHHRMTNFKFDPAHIRAPRSTSAKPPPANDTSDVIIFAPELFD
 2 DRR068691          100.0%  96.8%   FANELGGMELIASPIKERKTILRGGSYDFSAYRSYQKRHHRMTNFKFDPAHIRAPRSTFAKPLPANDTSDVIIFAPELFD
 3 DRR006265          100.0%  97.2%   FANELGGMELITSPIKERKTILRGGSYDFSAYRSYQKRHHRMTNFKFDPAHIRAPRSTSAKPLPANDTSDIIISAPELFD
 4 DRR039989           99.9%  97.1%   FANELGGMELITSPIKERKTILRGGSYDFSAYRSYQKRHHRMTNFKFDPAHIRAPRSTSAKPLPANDTSDVIISAPELFD
 5 DRR079253           99.9%  97.2%   FANELGGMELITSPIKERKTILRGGSYDFSAYRSYQKRHHRMTNFKFDPAHIRAPRSTSAKPLPANDTSDIIISAPELFD
 6 SRR6324419-24      100.0%  95.4%   FANELRGMEPITSPIKERKTILRGGSYHFSAFRSYQQKHHRMTNFKFDPAHTRAPRSASAKPLPANDASDIITFAPELFD
 7 SRR11781560-1      100.0%  95.5%   FANELRGMEPITSPIKERKTILRGGSYHFSAFRRKLPKHHRITNFKFDPAHTRAPRSASAKPLPANDTSDVIIFAPELFD
 8 SRR5458683         100.0%  97.2%   FANELGGMELITSPIKERKTILRGGSYDFSAYRSYQKRHHRMTNFKFDPAHIRAPRSTSAKPLPANDTSDIIISAPELFD
 9 DRR023337+40       100.0%  97.1%   FANELGGMELITSPIKERKTILRGGSYDFSAYRSYQKRHHRMTNFKFDPAHIRAPRSTSAKPLPANDTSDIIISAPELFD
10 SRR1333837-38      100.0%  97.2%   FANELGGMELITSPIKERKTILRGGSYDFSAYRSYQKRHHRMTNFKFDPAHIRAPRSTSAKPLPANDTSDIIISAPELFD
11 DRR241076          100.0%  98.5%   FANELGGMELITSPIKERKTILRGGSYDFSAYRSYQKRHHRMTNFKFDPAHIRAPRSTSAKPLPANDTSDVIIFAPELFD
12 SRR17258733        100.0%  97.3%   FANELGGMELITSPIKERKTILRGGSYDFSAYRSYQKRHHRMTNFKFDPAHIRAPRSTSAKPLPANDTSDIIISAPELFD
13 SRR24542361        100.0%  96.1%   FANELGGMELITSPIKERKTILRGGSYDFSAFRSHQKRHHRMTNFKFDPAHIRAPRPTSAKPLPANDTSDIIIFAPELFD
14 SRR17258732        100.0%  95.7%   FANELGGMELITSPIKERKTILRGGSYDFSAFRSHQKRHHRMTNFKFDPAHIRAPRPASAKPLPANDTSDIIIFAPELFD
15 SRR9685281         100.0%  97.2%   FANELGGMELITSPIKERKTILRGGSYDFSAYRSYQKRHHRMTNFKFDPAHIRAPRSTSAKPLPANDTSDIIISAPELFD
16 DRR035925          100.0%  99.9%   FANELGGMELITSPIKERKTILRGGSYDFSAYRSYQKRHHRMTNFKFDPAHIRAPLSTSAKPPPANDTSDVIIFAPELFD
17 AB186123.1          99.9%  97.5%   FANELGGMELITSPIKERKTILRGGSYDFSAYRSYQKRHHRMTNFKFDPAHTRAPRSTSAKPLPANDTSDIIIFAPELFD
18 KJ433990           100.0%  97.5%   FANELGGMELITSPIKERKTILRGGSYDFSAYRSYQKRHHRMTNFKFDPAHIRAPRSTFAKPLPANDTSDIIIFAPELFD
19 AB624361.1         100.0%  97.3%   FANELGGMELITSPIKERKTILRGGSYDFSAYRSYQKRHHRMTNFKFDPAHIRAPRSTSAKPLPANDTSDVIIFAPELFD
   consensus/100%                     FANELtGME.IsSPIKERKTILRGGSYcFSAaRph..+HHRhTNFKFDPAHhRAPhss.AKP.PANDsSDlIh.APELFD
   consensus/90%                      FANELtGME.ITSPIKERKTILRGGSYcFSAaRSaQp+HHRMTNFKFDPAHhRAPRss.AKP.PANDTSDlII.APELFD
   consensus/80%                      FANELGGMELITSPIKERKTILRGGSYDFSAaRSYQKRHHRMTNFKFDPAHIRAPRSTSAKPLPANDTSDlII.APELFD
   consensus/70%                      FANELGGMELITSPIKERKTILRGGSYDFSAYRSYQKRHHRMTNFKFDPAHIRAPRSTSAKPLPANDTSDlII.APELFD

                          cov    pid 1201      .         .         .         .         :         .         .        . 1280
 1 DRR068690          100.0% 100.0%   LQSSAIPRLDTHHLPETRRPLHFDIPSSLPTKADISGAEPTDTSIEPVYPGCDYKTVAALMMEPRDPDSLEIRHNGEFSN
 2 DRR068691          100.0%  96.8%   LQSSAIPRLDTHHLPETRRPLHFDIPSSLPTKVDISSVEPTDTAIEPVYPGCDYKTVAALMMEPRDPDSLEIRHNGEFSN
 3 DRR006265          100.0%  97.2%   LQSSAIPRLDTHHLPETRRPLHFDIPSSLPTKVDISSVEPTDTAIEPVYPGCDYKTVAALMMEPRDPDSLEIRHKGEFSN
 4 DRR039989           99.9%  97.1%   LQSSAIPRLDTHHLPETRRPLHFDIPSSLPTKVDISSVEPTDTAIEPVYPGCDYKTVAALMMEPRDPDSLEIRHKGEFSN
 5 DRR079253           99.9%  97.2%   LQSSAIPRLDTHHLPETRRPLHFDIPSSLPTKVDISSVEPTDTAIEPVYPGCDYKTVAALMMEPRDPDSLEIRHKGEFSN
 6 SRR6324419-24      100.0%  95.4%   LQSSAIPRLDTHHLPETRRPLHFDIPSSLPTKVDISSVEPTDTAIEPVYPGCDYKTVAALMMEPRNPDSLEIRHKGEFSN
 7 SRR11781560-1      100.0%  95.5%   LQSSAIPRLDTHHLPETRRPLHFDIPSSLPTKVDISSVEPTDTAIEPVYPGCDYKTVAALMMEPRDPDSLEIRHDGEFSN
 8 SRR5458683         100.0%  97.2%   LQSSAIPRLDTHHLPETRRPLHFDIPSSLPTKVDISSVEPTDTAIEPVYPGCDYKTVAALMMEPRDPDSLEIRHKGEFSN
 9 DRR023337+40       100.0%  97.1%   LQSSAIPRLDTHHLPETRRPLHFDIPSSLPTKVDISSVEPTDTAIEPVYPGCDYKTVAALMMEPRDPDSLEIRHKGEFSN
10 SRR1333837-38      100.0%  97.2%   LQSSAIPRLDTHHLPETRRPLHFDIPSSLPTKVDISSVEPTDTAIEPVYPGCDYKTVAALMMEPRDPDSLEIRHKGEFSN
11 DRR241076          100.0%  98.5%   LQSSAIPRLDTHHLPETRRPLHFDIPSSLPTKADISGAEPTDTSIEPVYPGCDYKTVAALMMEPRDPDSLEIRHNGEFSN
12 SRR17258733        100.0%  97.3%   LQSSAIPRLDTHHLPETRRPLHFDIPSSLPTKVDISSVEPTDTAIEPVYPGCDYKTVAALMMEPRDPDSLEIRHKGEFSN
13 SRR24542361        100.0%  96.1%   LQSSAIPRLDTHHLPETRRPLHFDIPSSLPTKVDISSVESTDTAIEPVYPGCDYKTVAALMMDPRDPDSLEIRHKGEFSN
14 SRR17258732        100.0%  95.7%   LQSSAIPRLDTHHIPETRRPLHFDIPSSLPTKVDISSVESTDTAIEPVYPGCDYKTVAALMMDPRDPDSLEIRHKGEFSN
15 SRR9685281         100.0%  97.2%   LQSSAIPRLDTHHLPETRRPLHFDIPSSLPTKVDISSVEPTDTAIEPVYPGCDYKTVAALMMEPRDPDSLEIRHKGEFSN
16 DRR035925          100.0%  99.9%   LQSSAIPRLDTHHLPETRRPLHFDIPSSLPTKADISGAEPTDTSIEPVYPGCDYKTVAALMMEPRDPDSLEIRHNGEFSN
```

```
17 AB186123.1       99.9%  97.5%      LQSSAIPRLDTHHLPETRRPLHFDIPSSLPTKVDISSAEPTDTAIEPVYPGCDYKTVAALMMEPRDPDSLEIRHKGEFSN
18 KJ433990        100.0%  97.5%      LQSSAIPRLDTHHLPETRRPLHFDILSSLPTKVDISSVEPTDTAIEPVYPGCDYKTVAALMMEPRDPDSLEIRHKGEFSN
19 AB624361.1      100.0%  97.3%      LQSSAIPRLDTHHLPETRRPLHFDIPSSLPTKVDISSAEPTDTSIEPVYPGCDYKTVAALMMEPRDPDSLEIRHNGEFSN
   consensus/100%                     LQSSAIPRLDTHHlPETRRPLHFDI.SSLPTKsDISusEsTDTuIEPVYPGCDYKTVAALMM-PRsPDSLEIRHpGEFSN
   consensus/90%                      LQSSAIPRLDTHHLPETRRPLHFDIPSSLPTKsDISusEsTDTuIEPVYPGCDYKTVAALMM-PRDPDSLEIRHpGEFSN
   consensus/80%                      LQSSAIPRLDTHHLPETRRPLHFDIPSSLPTKVDISSsEPTDTuIEPVYPGCDYKTVAALMMEPRDPDSLEIRHpGEFSN
   consensus/70%                      LQSSAIPRLDTHHLPETRRPLHFDIPSSLPTKVDISSVEPTDTAIEPVYPGCDYKTVAALMMEPRDPDSLEIRHcGEFSN

                      cov    pid 1281         .         3         .         .         .         .         :         . 1360
 1 DRR068690       100.0% 100.0%      QFPWVDLPYENGAQTLSVIAPKHDSKYDPTLLSASIAKRLRFRPSFLPYRLSPSDEVLGTFLFSSLCRAYKRHPNHKVPF
 2 DRR068691       100.0%  96.8%      QFPWVDLPHENGAQTLSVIAPKHDSKNDPTLLSASIAKRLRFRPSFLPYRLSPSDEVLGTFLFSSLCRAYKRHPNHKVPF
 3 DRR006265       100.0%  97.2%      QFPWVDLPYENGAQTLSVIAPKHDSKYDPTLLSASIAKRLRFRPSFLPYRLSPSDEVLGTFLFSSLCRAYKRHPNHKVPF
 4 DRR039989        99.9%  97.1%      QFPWVDLPYENGAQTLSVIAPKHDSKYDPTLLSASIAKRLRFRPSLLPYRLSPSDEVLGTFLFSSLCRAYKRHPNHKVPF
 5 DRR079253        99.9%  97.2%      QFPWVDLPYENGAQTLSVIAPKHDSKYDPTLLSASIAKRLRFRPSFLPYRLSPSDEVLGTFLFSSLCRAYKRHPNHKVPF
 6 SRR6324419-24   100.0%  95.4%      QFPWVDLPYENGAQTLSVIAPKHDSKNDPTLLSASIAKRLRFRPSLLPYRLSPSDEVLGTFLFSSLCRAYKRHPNHKVPF
 7 SRR11781560-1   100.0%  95.5%      QFPWVDLHYENGAQTLSVIAPKHDSKNDPTLLSASIAKRLRFRPSLLPYRLSPSDEVLGTFLFSSLCRAYKRHPNHKVPF
 8 SRR5458683      100.0%  97.2%      QFPWVDLPYENGAQTLSVIAPKHDSKYDPTLLSASIAKRLRFRPSFLPYRLSPSDEVLGTFLFSSLCRAYKRHPNHKVPF
 9 DRR023337+40    100.0%  97.1%      QFPWVDLPYENGAQTLSVIAPKHDSKYDPTLLSASIAKRLRFRPSFLPYRLSPSDEVLGTFLFSSLCRAYKRHPNHKVPF
10 SRR1333837-38   100.0%  97.2%      QFPWVDLPYENGAQTLSVIAPKHDSKYDPTLLSASIAKRLRFRPSFLPYRLSPSDEVLGTFLFSSLCRAYKRHPNHKVPF
11 DRR241076       100.0%  98.5%      QFPWVDLPYENGAQTLSVIAPKHDSKHDPTLLSASIAKRLRFRPSYLPYRLSPSDEVLGTFLFSSLCRAYKRHPNHKVPF
12 SRR17258733     100.0%  97.3%      QFPWVDLPYENGAQTLSVIAPKHDSKYDPTLLSASIAKRLRFRPSFLPYRLSPSDEVLGTFLFSSLCRAYKRHPNHKVPF
13 SRR24542361     100.0%  96.1%      QFPWVDLPYENGAQTLSVIAPKHDSKYDPTLLSASIAKRLRFRPSFLPYRLSPSDEVLGTFLFSSLCRAYKRHPNHKVPF
14 SRR17258732     100.0%  95.7%      QFPWVDLPYENGAQTLSVIAPKHDSKNDPTLLSASIAKRLRFRPSFLPYRLSPSDEVLGTFLFSSLCRAYKRHPNHKVPF
15 SRR9685281      100.0%  97.2%      QFPWVDLPYENGAQTLSVIAPKHDSKYDPTLLSASIAKRLRFRPSFLPYRLSPSDEVLGTFLFSSLCRAYKRHPNHKVPF
16 DRR035925       100.0%  99.9%      QFPWVDLPYENGAQTLSVIAPKHDSKYDPTLLSASIAKRLRFRPSFLPYRLSPSDEVLGTFLFSSLCRAYKRHPNHKVPF
17 AB186123.1       99.9%  97.5%      QFPWVDLPYENGAQTLSVIAPKHDSKYDPTLLSASIAKRLRFRPSFLPYRLSPSDEVLGTFLFSSLCRAYKRHPNHKVPF
18 KJ433990        100.0%  97.5%      QFPWVDLPHENGAQTLSVIAPKHDSKYDPTLLSASIAKRLRFRPSFLPYRLSPSDEVLGTFLFSSLCRAYKRHPNHKVPF
19 AB624361.1      100.0%  97.3%      QFPWVDLPYENGAQTLSVIAPKHDSKYDPTLLSASIAKRLRFRPSFLPYRLSPSDEVLGTFLFSSLCRAYKRHPNHKVPF
   consensus/100%                     QFPWVDL.aENGAQTLSVIAPKHDSK.DPTLLSASIAKRLRFRPShLPYRLSPSDEVLGTFLFSSLCRAYKRHPNHKVPF
   consensus/90%                      QFPWVDLPaENGAQTLSVIAPKHDSK.DPTLLSASIAKRLRFRPShLPYRLSPSDEVLGTFLFSSLCRAYKRHPNHKVPF
   consensus/80%                      QFPWVDLPYENGAQTLSVIAPKHDSK.DPTLLSASIAKRLRFRPSaLPYRLSPSDEVLGTFLFSSLCRAYKRHPNHKVPF
   consensus/70%                      QFPWVDLPYENGAQTLSVIAPKHDSKYDPTLLSASIAKRLRFRPSFLPYRLSPSDEVLGTFLFSSLCRAYKRHPNHKVPF

                      cov    pid 1361         .         .         .         4         .         .         .         . 1440
 1 DRR068690       100.0% 100.0%      QPDLFVECINLNEYSQLSNKTQAVIQANANRSDPDWRYTAVRIFAKTQHKINEGSIFGPWKACQTLALMHDAIVLIFGPI
 2 DRR068691       100.0%  96.8%      QPDLFVECINLNEYSQLSNKTQAVIQANANRSDPDWRYTAVRIFSKTQHKINEGSIFGPWKACQTLALMHDAIVLIFGPI
 3 DRR006265       100.0%  97.2%      QPDLFVECINLNEYSQLSNKTQAVIQANANRSDPDWRYTAVRIFSKTQHKINEGSIFGPWKACQTLALMHDAIVLIFGPI
 4 DRR039989        99.9%  97.1%      QPDLFVECINLNEYSQLSNKTQAVIQANANRSDPDWRYTAVRIFSKTQHKINEGSIFGPWKACQTLALMHDAIVLIFGPI
 5 DRR079253        99.9%  97.2%      QPDLFVECINLNEYSQLSNKTQAVIQANANRSDPDWRYTAVRI-SKTQHKINEGSIFGPWKACQTLALMHDAIVLIFGPI
 6 SRR6324419-24   100.0%  95.4%      QPDLFVECINLNEYSQLSNKTQAVIQANANRSDPDWRYTAVRIFSKTQHKINEGSIFGPWKACQTLALMHDAIVLIFGPI
 7 SRR11781560-1   100.0%  95.5%      QPDLFVECINLNEYSQLSNKTQAVIQANANRSDPDWRYTAVRIFSKTQHKINEGSIFGPWKACQTLALMHDVIVLIFGPI
 8 SRR5458683      100.0%  97.2%      QPDLFVECINLNEYSQLSNKTQAVIQANANRSDPDWRYTAVRIFSKTQHKINEGSIFGPWKACQTLALMHDAIVLIFGPI
 9 DRR023337+40    100.0%  97.1%      QPDLFVECINLNEYSQLSNKTQAVIQANANRSDPDWRYTAVRIFSKTQHKINEGSIFGPWKACQTLALMHDAIVLIFGPI
10 SRR1333837-38   100.0%  97.2%      QPDLFVECINLNEYSQLSNKTQAVIQANANRSDPDWRYTAVRIFSKTQHKINEGSIFGPWKACQTLALMHDAIVLIFGPI
```

```
11 DRR241076        100.0%  98.5%     QPDLFVECINLNEYSQLSNKTQAVIQANANRSDPDWRYTAVRIFAKTQHKINEGSIFGPWKACQTLALMHDAIVLIFGPI
12 SRR17258733      100.0%  97.3%     QPDLFVECINLNEYSQLSNKTQAVIQANANRSDPDWRYTAVRIFSKTQHKINEGSIFGPWKACQTLALMHDAIVLIFGPI
13 SRR24542361      100.0%  96.1%     QPDLFVECINLNEYSQLSNKTQAVIQANANRSDPDWRYTAVRIFSKTQHKINEGSIFGPWKACQTLALMHDAIVLIFGPI
14 SRR17258732      100.0%  95.7%     QPDLFVECINLNEYSQLSNKTQAVIQANANRSDPDWRYTAVRIFSKTQHKINEGSIFGPWKACQTLALMHDAIVLIFGPI
15 SRR9685281       100.0%  97.2%     QPDLFVECINLNEYSQLSNKTQAVIQANANRSDPDWRYTAVRIFSKTQHKINEGSIFGPWKACQTLALMHDAIVLIFGPI
16 DRR035925        100.0%  99.9%     QPDLFVECINLNEYSQLSNKTQAVIQANANRSDPDWRYTAVRIFAKTQHKINEGSIFGPWKACQTLALMHDAIVLIFGPI
17 AB186123.1        99.9%  97.5%     QPDLFVECINLNEYSQLSNKTQAVIQANANRSDPDWRYTAVRIFSKTQHKINEGSIFGPWKACQTLALMHDAIVLIFGPI
18 KJ433990         100.0%  97.5%     QPDLFVECINLNEYSQLSNKTQAVIQANANRSDPDWRYTAVRIFSKTQHKINEGSIFGPWKACQTLALMHDAIVLIFGPI
19 AB624361.1       100.0%  97.3%     QPDLFVECINLNEYSQLSNKTQAVIQANANRSDPDWRYTAVRIFAKTQHKINEGSIFGPWKACQTLALMHDAIVLIFGPI
   consensus/100%                     QPDLFVECINLNEYSQLSNKTQAVIQANANRSDPDWRYTAVRI.uKTQHKINEGSIFGPWKACQTLALMHDsIVLIFGPI
   consensus/90%                      QPDLFVECINLNEYSQLSNKTQAVIQANANRSDPDWRYTAVRIFuKTQHKINEGSIFGPWKACQTLALMHDAIVLIFGPI
   consensus/80%                      QPDLFVECINLNEYSQLSNKTQAVIQANANRSDPDWRYTAVRIFuKTQHKINEGSIFGPWKACQTLALMHDAIVLIFGPI
   consensus/70%                      QPDLFVECINLNEYSQLSNKTQAVIQANANRSDPDWRYTAVRIFSKTQHKINEGSIFGPWKACQTLALMHDAIVLIFGPI

                        cov    pid 1441         :         .         .         .         .         5         .         . 1520
 1 DRR068690        100.0% 100.0%     KKYQRVFDSKDRPPNLFVYGGQTPYDLSRFASSHLKPLQIHVCNDYSAFDQSQHGEAVVLERKKMERLSIPSHLIDLHCY
 2 DRR068691        100.0%  96.8%     KKYQRVFDSKDRPPNLFVYGGQTPYDLSRFASSHLKPLQIHVCNDYSAFDQSQHGEAVVLERKKMERLSIPSHLIDLHCY
 3 DRR006265        100.0%  97.2%     KKYQRVFDSKDRPPNLFVYGGQTPYDLSRFAASHLKPLQIHVCNDYSAFDQSQHGEAVVLERKKMERLSIPSHLIDLHCY
 4 DRR039989         99.9%  97.1%     KKYQRVFDSKDRPPNLFVYGGQTPYDLSRFAASHLKPLQIHVCNDYSAFDQSQHGEAVVLERKKMERLSIPSHLIDLHCY
 5 DRR079253         99.9%  97.2%     KKYQRVFDSKDRPPNLFVYGGQTPYDLSRFASSHLKPLQIHVCNDYSAFDQSQHGEAVVLERKKMERLSIPSHLIDLHCY
 6 SRR6324419-24    100.0%  95.4%     KKYQRVFDSKDRPPNLFVYGGQTPYDLSRFASSHLKPLQIHVCNDYSAFDQSQHGEAVVLERKKMERLSIPSHLIDLHCY
 7 SRR11781560-1    100.0%  95.5%     KKYQRVFDSKDRPPNLFVYGGQTPYDLSRFASSHLKPLQIHVCNDYSAFDQSQYGEAVVLERKKMERLSIPSHLIDLHCY
 8 SRR5458683       100.0%  97.2%     KKYQRVFDSKDRPPNLFVYGGQTPYDLSRFAASHLKPLQIHVCNDYSAFDQSQHGEAVVLERKKMERLSIPSHLIDLHCY
 9 DRR023337+40     100.0%  97.1%     KKYQRVFDSKDRPPNLFVYGGQTPYDLSRFAASHLKPLQIHVCNDYSAFDQSQHGEAVVLERKKMERLSIPSHLIDLHCY
10 SRR1333837-38    100.0%  97.2%     KKYQRVFDSKDRPPNLFVYGGQTPYDLSRFASSHLKPLQIHVCNDYSAFDQSQHGEAVVLERKKMERLSIPSHLIDLHCY
11 DRR241076        100.0%  98.5%     KKYQRVFDSKDRPPNLFVYGGQTPYDLSRFASSHLKPLQIHVCNDYSAFDQSQHGEAVVLERKKMERLSIPSHLIDLHCY
12 SRR17258733      100.0%  97.3%     KKYQRVFDSKDRPPNLFVYGGQTPYDLSRFASSHLKPLQIHVCNDYSAFDQSQHGEAVVLERKKMERLSIPSHLIDLHCY
13 SRR24542361      100.0%  96.1%     KKYQRVFDSKDRPPNLFVYGGQTPYDLSRFASSHLKPLQIHVCNDYTAFDQSQHGEAVVLERKKMERLSIPSHLIDLHCY
14 SRR17258732      100.0%  95.7%     KKYQRIFDSKDRPPNLFVYGGQTPYDLSRFASSHLKPLQIHVCNDYTAFDQSQHGEAVVLERKKMERLSIPSHLIDLHCY
15 SRR9685281       100.0%  97.2%     KKYQRVFDSKDRPPNLFVYGGQTPYDLSRFAASHLKPLQIHVCNDYSAFDQSQHGEAVVLERKKMERLSIPSHLIDLHCY
16 DRR035925        100.0%  99.9%     KKYQRVFDSKDRPPNLFVYGGQTPYDLSRFASSHLKPLQIHVCNDYSAFDQSQHGEAVVLERKKMERLSIPSHLIDLHCY
17 AB186123.1        99.9%  97.5%     KKYQRVFDSKDRPPNLFVYGGQTPYDLSRFASSHLKPLQIHVCNDYSAFDQSQHGEAVVLERKKMERLSIPSHLIDLHCY
18 KJ433990         100.0%  97.5%     KKYQRVFDSKDRPPNLFVYGGQTPYDLSRFASSHLKPLQIHVCNDYSAFDQSQHGEAVVLERKKMERLSIPSHLIDLHCY
19 AB624361.1       100.0%  97.3%     KKYQRVFDSKDRPPNLFVYGGQTPYDLSRFASSHLKPLQIHVCNDYSAFDQSQHGEAVVLERKKMERLSIPSHLIDLHCY
   consensus/100%                     KKYQRlFDSKDRPPNLFVYGGQTPYDLSRFAuSHLKPLQIHVCNDYoAFDQSQaGEAVVLERKKMERLSIPSHLIDLHCY
   consensus/90%                      KKYQRVFDSKDRPPNLFVYGGQTPYDLSRFAuSHLKPLQIHVCNDYoAFDQSQHGEAVVLERKKMERLSIPSHLIDLHCY
   consensus/80%                      KKYQRVFDSKDRPPNLFVYGGQTPYDLSRFAuSHLKPLQIHVCNDYSAFDQSQHGEAVVLERKKMERLSIPSHLIDLHCY
   consensus/70%                      KKYQRVFDSKDRPPNLFVYGGQTPYDLSRFASSHLKPLQIHVCNDYSAFDQSQHGEAVVLERKKMERLSIPSHLIDLHCY

                        cov    pid 1521         .         .         :         .         .         .         .        6 1600
 1 DRR068690        100.0% 100.0%     IKTNITSQFGPLTCMRLTGEPGTYDDNTDYNIAVIYSEYDITTQTVFVSGDDSLVSPPPPAHPDWDAVSPLLSLTFKKEF
 2 DRR068691        100.0%  96.8%     IKTNITSQFGPLTCMRLTGEPGTYDDNTDYNIAVIYSEYDITTQTVFVSGDDSLVSPPPPAHPDWDAIAPLLSLTFKKEF
 3 DRR006265        100.0%  97.2%     IKTNITSQFGPLTCMRLTGEPGTYDDNTDYNIAVIYSEYDITTQTVFVSGDDSLVSPPPPAHPDWDAVSPLLSLTFKKEF
 4 DRR039989         99.9%  97.1%     IKTNITSQFGPLTCMRLTGEPGTYDDNTDYNIAVIYSEYDITTQTVFVSGDDSLVSPPPPAHPDWDAVSPLLSLTFKKEF
```

```
 5 DRR079253        99.9%  97.2%       IKTNITSQFGPLTCMRLTGEPGTYDDNTDYNIAVIYSEYDITTQTVFVSGDDSLVSPPPPAHPDWDAVSPLLSLTFKKEF
 6 SRR6324419-24   100.0%  95.4%       IKTNITSQFGPLTCMRLTGEPGTYDDNTDYNIAVIYSEYDITTQTVFVSGDDSLVSPPPPAHPDWDAISPLLSLTFKKEF
 7 SRR11781560-1   100.0%  95.5%       IKTNITSQFGPLTCMRLTGEPGTYDDNTDYNIAVIYSEYDITTQTVFVSGDDSLVSPPPPAHPDWDAISPLLSLTFKKEF
 8 SRR5458683      100.0%  97.2%       IKTNITSQFGPLTCMRLTGEPGTYDDNTDYNIAVIYSEYDITTQTVFVSGDDSLVSPPPPAHPDWDAVSPLLSLTFKKEF
 9 DRR023337+40    100.0%  97.1%       IKTNITSQFGPLTCMRLTGEPGTYDDNTDYNIAVIYSEYDITTQTVFVSGDDSLVSPPPPAHPDWDAVSPLLSLTFKKEF
10 SRR1333837-38   100.0%  97.2%       IKTNITSQFGPLTCMRLTGEPGTYDDNTDYNIAVIYSEYDITTQTVFVSGDDSLVSPPPPAHPDWDAVSPLLSLTFKKEF
11 DRR241076       100.0%  98.5%       IKTNITSQFGPLTCMRLTGEPGTYDDNTDYNIAVIYSEYDITTQTVFVSGDDSLVSPPPPAHPDWDAVSPLLSLTFKKEF
12 SRR17258733     100.0%  97.3%       IKTNITSQFGPLTCMRLTGEPGTYDDNTDYNIAVIYSEYDITTQTVFVSGDDSLVSPPPPAHPDWDAVSPLLSLTFKKEF
13 SRR24542361     100.0%  96.1%       IKTNITSQFGPLTCMRLTGEPGTYDDNTDYNIAVIYSEYDITTQTVFVSGDDSLVSPPPPAHPDWDAVSPLLSLTFKKEF
14 SRR17258732     100.0%  95.7%       IKTNITSQFGPLTCMRLTGEPGTYDDNTDYNIAVIYSEYDITTQTVFVSGDDSLVSPPPPAHPDWDAVSPLLSLTFKKEF
15 SRR9685281      100.0%  97.2%       IKTNITSQFGPLTCMRLTGEPGTYDDNTDYNIAVIYSEYDITTQTVFVSGDDSLVSPPPPAHPDWDAVSPLLSLTFKKEF
16 DRR035925       100.0%  99.9%       IKTNITSQFGPLTCMRLTGEPGTYDDNTDYNIAVIYSEYDITTQTVFVSGDDSLVSPPPPAHPDWDAVSPLLSLTFKKEF
17 AB186123.1       99.9%  97.5%       IKTNITSQFGPLTCMRLTGEPGTYDDNTDYNIAVIYSEYDITTQTVFVSGDDSLVSPPPPAHPDWDAVSPLLSLTFKKEF
18 KJ433990        100.0%  97.5%       IKTNITSQFGPLTCMRLTGEPGTYDDNTDYNIAVIYSEYDITTQTVFVSGDDSLVSPPPPAHPDWDAVSPLLSLTFKKEF
19 AB624361.1      100.0%  97.3%       IKTNITSQFGPLTCMRLTGEPGTYDDNTDYNIAVIYSEYDITTQTVFVSGDDSLVSPPPPAHPDWDAVSPLLPLTFKKEF
   consensus/100%                      IKTNITSQFGPLTCMRLTGEPGTYDDNTDYNIAVIYSEYDITTQTVFVSGDDSLVSPPPPAHPDWDAluPLLsLTFKKEF
   consensus/90%                       IKTNITSQFGPLTCMRLTGEPGTYDDNTDYNIAVIYSEYDITTQTVFVSGDDSLVSPPPPAHPDWDAlSPLLSLTFKKEF
   consensus/80%                       IKTNITSQFGPLTCMRLTGEPGTYDDNTDYNIAVIYSEYDITTQTVFVSGDDSLVSPPPPAHPDWDAVSPLLSLTFKKEF
   consensus/70%                       IKTNITSQFGPLTCMRLTGEPGTYDDNTDYNIAVIYSEYDITTQTVFVSGDDSLVSPPPPAHPDWDAVSPLLSLTFKKEF

                    cov     pid  1601          .         .         .         .         :         .         .         . 1680
 1 DRR068690       100.0% 100.0%       VRNGLFCGYYLGPAGAIRAPRALLAKLVLALDDRTLPDKIASYLSEFVVGHSLGDAFWTLLPADQVIYQSALFDFFCREC
 2 DRR068691       100.0%  96.8%       VRHGLFCGYYLGPAGAIRAPRALLAKLVLALDDGTLPDKIASYLSEFVVGHSLGDAFWTLLPADQVIYQSALFDFFCREC
 3 DRR006265       100.0%  97.2%       VRNGLFCGYYLGPAGAIRAPRALLAKLVLALDDRTLPDKIASYLSEFVVGHSLGDAFWTLLPADQVIYQSALFDFFCREC
 4 DRR039989        99.9%  97.1%       VRNGLFCGYYLGPAGAIRAPRALLAKLVLALDDRTLPDKIASYLSEFVVGHSLGDAFWTLLPADQVIYQSALFDFFCREC
 5 DRR079253        99.9%  97.2%       VRNGLFCGYYLGPAGAIRAPRALLAKLVLALDDRTLPDKIASYLSEFVVGHSLGDAFWTLLPADQVIYQSALFDFFCREC
 6 SRR6324419-24   100.0%  95.4%       VRNGLFCGYYLGPAGAIRAPRALLAKLVLALDDGTLPDKIASYLSEFVVGHSLGDAFWTLLPADQVIYQSALFDFFCREC
 7 SRR11781560-1   100.0%  95.5%       VRNGLFCGYYLGPAGAIRAPRALLAKLVLALDDGTLPDKIASYLSEFVVGHSLGDAFWTLLPADQVIYQSALFDFFCREC
 8 SRR5458683      100.0%  97.2%       VRNGLFCGYYLGPAGAIRAPRALLAKLVLALDDRTLPDKIASYLSEFVVGHSLGDAFWTLLPADQVIYQSALFDFFCREC
 9 DRR023337+40    100.0%  97.1%       VRNGLFCGYYLGPAGAIRAPRALLTKLVLALDDRTLPDKIASYLSEFVVGHSLGDAFWTLLPADQVIYQSALFDFFCREC
10 SRR1333837-38   100.0%  97.2%       VRNGLFCGYYLGPAGAIRAPRALLAKLVLALDDRTLPDKIASYLSEFVVGHSLGDAFWTLLPADQVIYQSALFDFFCREC
11 DRR241076       100.0%  98.5%       VRNGLFCGYYLGPAGAIRAPRALLAKLVLALDDRTLPDKIASYLSEFVVGHSLGDAFWTLLPADQVIYQSALFDFFCREC
12 SRR17258733     100.0%  97.3%       VRNGLFCGYYLGPAGAIRAPRALLAKLVLALDDRTLPDKIASYLSEFVVGHSLGDAFWTLLPADQVIYQSALFDFFCREC
13 SRR24542361     100.0%  96.1%       VRNGLFCGYYLGPAGAIRAPRALLAKLVLALDDGTLSDKIASYLSEFVVGHSLGDAFWTLLPADQVIYQSALFDFFCREC
14 SRR17258732     100.0%  95.7%       VRNGLFCGYYLGPAGAIRAPRALLAKLVLALDDGTLSDKIASYLSEFVVGHSLGDAFWTLLPADQVIYQSALFDFFCREC
15 SRR9685281      100.0%  97.2%       VRNGLFCGYYLGPAGAIRAPRALLAKLVLALDDRTLPDKIASYLSEFVVGHSLGDAFWTLLPADQVIYQSALFDFFCREC
16 DRR035925       100.0%  99.9%       VRNGLFCGYYLGPAGAIRAPRALLAKLVLALDDRTLPDKIASYLSEFVVGHSLGDAFWTLLPADQVIYQSALFDFFCREC
17 AB186123.1       99.9%  97.5%       VRNGLFCGYYLGPAGAIRAPRALLAKLVLALDDRTLPDKIASYLSEFVVGHSLGDAFWTLLPADQVIYQSALFDFFCREC
18 KJ433990        100.0%  97.5%       VRNGLFCGYYLGPAGAIRAPRALLAKLVLALDDRTLPDKIASYLSEFVVGHSLGDAFWTLLPADQVIYQSALFDFFCREC
19 AB624361.1      100.0%  97.3%       VRNGLFCGYYLGPAGAIRAPRALLAKLVLALDDRTLPDKIASYLSEFVVGHSLGDAFWTLLPADQVIYQSALFDFFCREC
   consensus/100%                      VRpGLFCGYYLGPAGAIRAPRALLsKLVLALDDtTLsDKIASYLSEFVVGHSLGDAFWTLLPADQVIYQSALFDFFCREC
   consensus/90%                       VRNGLFCGYYLGPAGAIRAPRALLAKLVLALDDtTLsDKIASYLSEFVVGHSLGDAFWTLLPADQVIYQSALFDFFCREC
   consensus/80%                       VRNGLFCGYYLGPAGAIRAPRALLAKLVLALDDtTLPDKIASYLSEFVVGHSLGDAFWTLLPADQVIYQSALFDFFCREC
   consensus/70%                       VRNGLFCGYYLGPAGAIRAPRALLAKLVLALDDRTLPDKIASYLSEFVVGHSLGDAFWTLLPADQVIYQSALFDFFCREC
```

```
                          cov    pid 1681        .         7         .         .         .         .         :         ] 1760
 1 DRR068690          100.0% 100.0%     SKEQKLALKIGEVPELLCQTMLSLGFSWLSRPLYALLDRTARLRLLARTRFAPLFSDPSVEGVLQLDF------------
 2 DRR068691          100.0%  96.8%     SKEQKLALKIGEVPEHLCQTMLSLGFSWLSRPLYALLDRTARLRLLARTRFAPLFSDPSVEGVLQLDF------------
 3 DRR006265          100.0%  97.2%     SKEQKLALKIGEVPEHLCQTMLSLGFSWLSRPLYALLDRTARLRLLARTRFAPLFSDPSVEGVLQLDF------------
 4 DRR039989           99.9%  97.1%     SKEQKLALKIGEVPEHLCQTMLSLGFSWLSRPLYALLDRTARLRLLARTRFAPLFSDPSVEGVLQLDF------------
 5 DRR079253           99.9%  97.2%     SKEQKLALKIGEVPEHLCQTMLSLGFSWLSRPLYALLDRTARLRLLARTRFAPLFSDPSVEGVLQLDF------------
 6 SRR6324419-24      100.0%  95.4%     SKEQKLALKIGEVPEHLCQTMLSLGFSWLSRPLYALLDRTARLRLLARTRFAPLFSDPSAESVLQLDF------------
 7 SRR11781560-1      100.0%  95.5%     SKEQKLALKIGEVPEHLCQTMLSLGFSWLSRPLYALLDRTARLRLLARTRFAPLFSDPSAEGVLQLDF------------
 8 SRR5458683         100.0%  97.2%     SKEQKLALKIGEVPEHLCQTMLSLGFSWLSRPLYALLDRTARLRLLARTRFAPLFSDPSVEGVLQLDF------------
 9 DRR023337+40       100.0%  97.1%     SKEQKLALKIGEVPEHLCQTMLSLGFSWLSRPLYALLDRTARLRLLARTRFAPLFSDPSVEGVLQLDF------------
10 SRR1333837-38      100.0%  97.2%     SKEQKLALKIGEVPEHLCQTMLSLGFSWLSRPLYALLDRTARLRLLARTRFAPLFSDPSVEGVLQLDF------------
11 DRR241076          100.0%  98.5%     SKEQKLALKIGEVPELLCQTMLSLGFSWLSRPLYALLDRTARLRLLARTRFAYLFSDPSVEGVLQLDF------------
12 SRR17258733        100.0%  97.3%     SKEQKLALKIGEVPEHLCQTMLSLGFSWLSRPLYALLDRTARLRLLARTRFAPLFSDPSVEGVLQLDF------------
13 SRR24542361        100.0%  96.1%     SKEQKLALKIGEVPEHLCQKMLSLGFSWLSHPLYALLDRTARLRLLARTRFAPLFSDPSVEGVLQLDF------------
14 SRR17258732        100.0%  95.7%     SKEQKLALKIGEVPEHLCQKMLSLGFSWLSHPLYALLDRTARLRLLARTRFAPLFSDPSVEGVLQLDF------------
15 SRR9685281         100.0%  97.2%     SKEQKLALKIGEVPEHLCQTMLSLGFSWLSRPLYALLDRTARLRLLARTRFAPLFSDPSVEGVLQLDF------------
16 DRR035925          100.0%  99.9%     SKEQKLALKIGEVPELLCQTMLSLGFSWLSRPLYALLDRTARLRLLARTRFAPLFSDPSVEGVLQLDF------------
17 AB186123.1          99.9%  97.5%     SKEQKLALKIGEVPEHLCQTMLSLGFSWLSRPLYALLDRTARLRLLARTRFAPLFSDPSVEGVLQLDF------------
18 KJ433990           100.0%  97.5%     SKEQKLALKIGEVPEHLCQTMLSLGFSWLSRPLYALLDRTARLRLLARTRFAPLFSDPSVEGVLQLDF------------
19 AB624361.1         100.0%  97.3%     SKEQKLALKIGEVPELLCQTMLSLGFSWLSRPLYALLDRTARLRLLARTRFAPPLFRSFCRGCFATGFLMTYSWKKLCLC
   consensus/100%                       SKEQKLALKIGEVPEhLCQpMLSLGFSWLS+PLYALLDRTARLRLLARTRFA..h.cs.scushthsF............
   consensus/90%                        SKEQKLALKIGEVPEhLCQpMLSLGFSWLS+PLYALLDRTARLRLLARTRFAPLFSDPSsEGVLQLDF............
   consensus/80%                        SKEQKLALKIGEVPEhLCQTMLSLGFSWLSRPLYALLDRTARLRLLARTRFAPLFSDPSVEGVLQLDF............
   consensus/70%                        SKEQKLALKIGEVPEHLCQTMLSLGFSWLSRPLYALLDRTARLRLLARTRFAPLFSDPSVEGVLQLDF............
```
